# Flot2 acts as a novel mediator of podocyte injury in proteinuric kidney disease

**DOI:** 10.7150/ijbs.78945

**Published:** 2023-01-01

**Authors:** Chunping Yu, Hong Zhang, Shuangxin Liu, Ruizhao Li, Xingchen Zhao, Yuanhan Chen, Zhuo Li, Jianchao Ma, Wenjian Wang, Zhiming Ye, Xinling Liang, Li Zhang, Wei Shi

**Affiliations:** 1 Department of Nephrology, Guangdong Provincial People's Hospital, Guangdong Academy of Medical Sciences, 106 Zhongshan No. 2 Road, Guangzhou, 510080, China.; 2 State Key Laboratory of Oncology in South China, Collaborative Innovation Center for Cancer Medicine, Sun Yat-sen University Cancer Center, Guangzhou, P. R. China.; 3 Department of Nephrology, Gaozhou People's Hospital, Gaozhou, P. R. China.

**Keywords:** Lipid raft, flot2, podocyte, slit diaphragm protein, proteinuria, chronic kidney disease

## Abstract

Podocyte injury is a common hallmark of chronic kidney disease (CKD). The podocin-nephrin complex localized in lipid rafts of podocyte is vital to reduce podocyte injury and proteinuria, however, the mechanism underlying its localization remains unclear. This study uncovers an important role of Flot2 in stabilizing the podocin-nephrin complex localized in lipid rafts. We first confirmed that Flot2 was expressed in podocyte and demenstrated that podocyte-specific Flot2 deletion worsen albuminuria, podocyte injury and glomerular pathology in LPS/ADR-induced nephropathy mouse models. Meanwhile, podocyte injury, albuminuria and pathologic aberrance were prevented in podocyte-specific Flot2 overexpression transgenic mice when challenged with LPS or ADR. Further found that Flot2 was vital to recruit podocin and nephrin into rafts and ameliorated podocyte injury. Flot2 and podocin directly interacted with each other via their SPFH domain. Meanwhile, we also showed that Flot-2 is a direct target of Krüppel-like factor (KLF15). Importanly, we observed that Flot2 was downregulated in renal biopsies from patients with podocytopathies and its expression negatively correlated with proteinuria and positively correlated with eGFR, indicating that Flot2 may be a novel therapeutic target for proteinuric kidney disease.

## Introduction

Chronic kidney disease (CKD) is a worldwide public health problem with increasing prevalence, poor outcomes, and high cost 1,2. Efficient therapeutic targets are therefore urgently needed to delay CKD progression and improve its prognosis.

Podocytes are highly differentiated epithelial cells located outside the glomerular basement membrane, which play a key role in maintaining selective glomerular filtration 3. The finger- like foot processes of podocytes intertwine to form the slit diaphragm (SD), which constitutes the most important component of the glomerular filtration barrier 4. The integrity of this filtration barrier is important to prevent the loss of protein into the urine (proteinuria) 5. Increasing numbers of disease-causing SD proteins have been identified, including nephrin, podocin, and CD2AP 6-13, and the SD has been shown to act not only as a filter to prevent proteinuria, but also as a complex signaling hub to divert chemical and mechanical stimuli to podocytes 4,5,14. Although many key components of the SD have now been identified, the ways in which the different components are organized at this specialized cell-cell junction remain unclear.

Extensive evidence has revealed that the SD has a lipid raft-like structure 5,9,15. Lipid rafts are specialized microdomains of the plasma membrane with a unique lipid composition, which are highly enriched in signal transduction molecules 16. Lipid raft microdomains in podocytes are critical for the dynamic functional organization of the SD 9,17. Podocin is a hairpin-like protein including cytoplasmic N- and C-termini with a transmembrane portion 18, which is expressed almost exclusively in SD in the kidney 6. Podocin is a lipid raft-associated protein that interacts with nephrin and CD2AP at the SD 18, and is believed to be important for the recruitment of nephrin to the lipid raft to augment nephrin signaling 19. However, the mechanism of podocin localization in the SD remain unclear.

Flotillin-2 (Flot2) is a major hydrophobic protein in lipid rafts. Flot2 was initially identified as gene products that were upregulated in optic nerve lesions and during axon regeneration in goldfish 20. Flot2 was subsequently found to play a role in neural differentiation 21,22. Flot2 protein acts as a scaffold for lipid rafts by forming homo- and hetero-oligomers 23,24, and was also reported to interact with cytoskeletal proteins such as tubulin and actin via their stomatin/prohibitin/flotillin/HflK/C(SPFH) domains 25,26. However, almost a decade after its discovery, the biologic function of Flot2 and its structural determinants for lipid-raft association in podocytes and proteinuric glomerular disease remain unknown. In this study, we aimed to identify and describe the functional role of Flot2 in recruiting SD proteins into rafts and in mediating podocyte injury and proteinuric glomerular disease.

## Methods

### Patients

The study of patients was conducted in accordance with the Second Helsinki Declaration and was approved by the Ethics Committee for Human Research of Guangdong Provincial People's Hospital (no.GDREC2013070H). Kidney biopsy samples were obtained from proteinuric patients with minimal change disease (MCD), focal segmental glomerulosclerosis (FSGS), Membranous nephropathy (MN), IgA nephropathy (IgAN), diabetic nephropathy (DN), and Control samples were obtained from the healthy kidney poles of individuals who underwent tumor nephrectomies without diabetes or renal disease. Written informed consent for kidney samples for research purposes was obtained from patients at the time of biopsy or before operation.

### Cell culture and treatments

The conditionally immortalized mouse podocyte cell line was kindly provided by Prof.Jochen Reiser (Rush University Medical Center, Chicago, IL, USA) and cultured as described previously 27. In brief, podocytes were maintained in RPMI 1640 (Corning, Manassas, USA) supplemented with 10% fetal bovine serum, 100U/ml penicillin, and 100mg/ml streptomycin. To propagate podocytes, cells were cultivated at 33 °C (permissive conditions), and the culture medium was supplemented with 10 U/ml mouse recombinant γ-interferon (ProSpec, Tany Technogene Ltd, Ness Ziona, Israel). To induce differentiation, podocytes were maintained on collagen type I at 37 °C without γ-interferon (nonpermissive conditions) for at least 10-14d. After differentiation, podocytes were confirmed by the identification of synaptopodin, a podocyte differentiation marker. Before stimulation, 10^6^cells were synchronized into quiescence by growing cells in serum-free RPMI-1640 medium for 24 h, and then treated with HG (30 mM glucose, Sigma-Aldrich, St.Louis, MO,USA), MA (5.3 mM glucose+24.7 mM D-mannitol (Sigma Aldrich, St. Louis, MO, USA) as an osmotic control, LPS (100 μg/ml, Sigma-Aldrich, St.Louis, MO, USA), ADR (0.25 μg/ml, Sigma-Aldrich, St. Louis, MO, USA) or small interfering RNA (siRNA) (50 nM) for 48 h.

### siRNA transfection and infection of adenovirus

The siRNA sequences targeting mouse Flot2, KLF4, KLF6 and KLF15 and control-siRNA were designed and synthesized respectively by RiboBio Co., Ltd. (Guangzhou, China). Transfection experiments were performed following transfection reagent, Lipofectamine 2000 (Invitrogen, Thermo Fisher Scientific, Waltham, MA) protocols. The sequences of these siRNAs used in this study and the efficiency of RNA interference were present in Supplementary Table S1 and Figure S1, respectively. To overexpress Flot2 or KLF15 in podocytes, the adenovirus containing ORF-Flot2 clone or ORF-KLF15 clone (Hanbio, Shanghai, China) were employed respectively according to the manufacturer's instructions. Cells were harvested for 48 hours after siRNA or adenovirus treatment for real-time quantitative PCR, immunofluorescence, and western blot analyses.

### Real-time quantitative-PCR

Total RNA of cultured podocytes under different conditions was extracted using Trizol RNA isolation system (Invitrogen, CA, USA) and reverse transcribed into cDNAs using the Prime ScriptTM RT regent Kit following the instructions provided by the manufacturer (Takara Biotechnology, Dalian, China), and then cDNAs were used for real-time PCR analysis by a Plantinum SYBR Green SuperMix-UDG kit (Takara Biotechnology, Dalian, China). Each reaction was amplified in triplicate. The 2^-ΔΔ^Ct method was used to quantify the relative expression levels of messenger RNA (mRNA). The primers used for qPCR are listed in Supplementary Table S2.

### Animal studies

All animal care and experiments were performed in accordance with the ARRIVE guidelines, and were approved by the Ethics Committee for animal research of Guangdong Provincial People's Hospital (no. GDREC2013070A). Animals had unrestricted access to water and food and were maintained on a 12 h light/ dark cycle in a specific pathogen-free environment. All Mice were anesthetized with pentobarbital sodium (60 mg/kg, intraperitoneally injected), and kidney tissue was collected.

### Generation of conditional podocyte-specific flot2 knockout mice

Flot2-floxed mice were generated by Shanghai Model Organisms Center, Inc., (Shanghai, China). Briefly, Exon 4 of murine flot2 was flanked with two loxp sequences, and a neomycin resistance cassette (Neo) flanked with two FRT sites was used for positive selection in mice embryonic stem cells (ES) clones. Positive ES cell clones were expanded and injected into *C57BL/6J* blastocysts to generate the chimeric offspring. The chimeric mice were mated with *C57BL/6J* mice to obtain the flot2 floxed heterozygous mouse flot2 fl/+. Floxed flot2 mice and podocyte-specific iCre recombinase, podocin-iCre (NPHS2-iCre) mice (a kind gift from Dr. Farhad Danesh, Bayor College of Medicine,Houston, TX, USA.) were crossed to generate podocyte-specific, flot2 knockout mice. Cre recombinase was induced by intraperitoneally injected with 1 mg of tamoxifen (Sigma Aldrich) per mice (8-week-old) for 10 consecutive days. Cre-mediated recombination resulted in deletion of the flot2 flanked sequence in the podocyte. We confirmed Flot2 deletion in the podocytes by genotyping and by immunostaining (Figure 2). All of the mouse lines were maintained on a *C57BL/6* background. Control mice were littermates of floxed flot2.

### Generation of conditional podocyte-specific flot2 knockin mice

Rosa26-flot2 mice were generated by Shanghai Model Organisms Center, Inc (Shanghai, China). Briefly, for the targeting vector, 9.5 kb fragment of murine flot2 containing cDNA was cloned, with a 1.0 kb genomic fragment upstream and a 4.3kb downstream from Rosa26 loci, respectively, acting as 5' arm and 3' homologous arm. A neomycin resistance cassette (Neo) flanked with two Loxp sites was used for positive selection in mice embryonic stem cells (ES) clones. Positive ES cell clones were expanded and injected into *C57BL/6J* blastocysts to generate the chimeric offspring. The chimeric mice were mated with *C57BL/6J* mice to obtain the flot2 knockin heterozygous mouse Rosa26-flot2. Rosa26-flot2 mice and NPHS2-i*Cre* mice were crossed to generate podocyte-specific, flot2 knockin mice. Cre recombinase was induced by intraperitoneally injected with 1 mg of tamoxifen (Sigma Aldrich) per mice (8-week-old) for 10 consecutive days, Cre-mediated recombination resulted in knockin the flot2 flanked sequence in the podocyte. We confirmed flot2 knockin in the podocytes by genotyping and by immunostaining (Figure 4). All of the mouse lines were maintained on a *C57BL/6* background. Control mice were littermates of Rosa26 flot2.

### Lipopolysaccharide (LPS)-induced nephropathy mice

Six-week-old Male *C57BL/6* mice were purchased from Center of Laboratory Animal Science of Guangdong, China. For the murine model of transient proteinuria (lipopolysaccharide (LPS)- treated *C57 BL/6* mice), 200μg LPS (L-2880, Sigma- Aldrich, St Louis, MO, USA) in a total volume of 100μl was intraperitoneally injected to eight-week old male *C57BL/6* mice and podocyte-specific flot2 knockout or knockin mice (*C57BL/6* at 8-9 weeks of age), respectively. Control group mice were intraperitoneally injected with equal volumes of sterile LPS-free PBS. Urine was collected before and 24 hours after LPS injection, and mice were sacrificed 24 hours after treatment.

### Adriamycin (ADR)-induced nephropathy mice

In the ADR model, eight-week-old Male* C57BL/6* mice and podocyte-specific flot2 knockout or knockin mice (*C57BL/6* at 8-9 weeks of age), were respectively administered ADR (18 mg/kg) intravenously by tail vein injection. Control group mice were i.p. injected with equal volumes of sterile saline. Urine was collected weekly to assess for albuminuria, and mice were sacrificed 12 weeks post-treatment.

### Diabetic mice model

The *C57BL/KsJ* db/db mice were obese and known to develop type 2 diabetes, followed by diabetic kidney disease. Eight-week-old male *C57BL/KsJ*-db/db mice and age-matched wild-type (*BKS*) mice were purchased from Model Animal Research Center of Nanjing University and housed in the animal center of Sun Yat-sen University Zhongshan School of Medicine, Guangzhou, China. Fasting blood glucose was measured in tail-vein blood, weekly, using a one touch ultra glucometer and Test Strip (Lifescan, Milpitas, CA, USA) after 6 h of fasting. Mice were sacrificed at 20 weeks.

### Urinary albumin and creatinine measurements

Mice urinary albumin and creatinine were measured using mouse albumin-specific ELISA (Bethyl Laboratories Inc, Montgomery, TX, USA) and creatinine kits (Cayman Chemical, Ann Arbor, MI, USA) respectively, according to the manufacturer's instructions. The results were presented as albumin/creatinine ratio (ACR, μg/mg).

### Immunofluorescent staining

Cultured podocytes planted on cover slides in six-well plates or frozen cryostat sections were fixed with 4% paraformaldehyde at room temperature(RT) for 20 min, permeabilized with 0.1% Triton X-100 for 10 min, then blocked with 5% bovine serum albumin for 30 min at RT before further incubated overnight at 4 °C with the following primary antibodies: goat anti-synaptopodin (Santa Cruz, 1:100), rabbit anti-Flot2 (Cell Signaling Technology, 1:50), Rabbit anti-podocin (Sigma,1:200).Then cultured podocytes or sections were washed three times with PBS for 5 min before the secondary antibodies (FITC-donkey anti-goat IgG 488, Life Technologies, USA, 1:250; goat anti-rabbit Alexa Fluor 555,Cell Signaling Technology, 1:200 were applied for 1 hour at RT in the dark. Culture podocytes were stained with phalloidin (Cytoskeleton,1:500) and Cholera toxin subunit B (CTXB, Life Technologies, USA, 1:500) for 30 min and then with DAPI(Sigma, St. Louis, MO, USA) for 5 min at RT. All images were taken using laser confocal microscopy (LCSM, Zeiss KS 400, Postfach, Germany).

### Histological assessment

Paraffin-embedded tissues were cut into 4-μm sections and stained with periodic acid-Schiff for light microscopic examination. In each group, more than 50 glomeruli were used and averaged for morphometric analysis. To quantify mesangial expansion, using Image-Pro Plus 6.0 (Media Cybernetics, Georgia Avenue, MD, USA) program, the area of the PAS-positive and nuclei-free material in the mesangium (mesangial area) was factored by the glomerular tuft area to arrive at the fraction of mesangial matrix. Sections were coded and read by an observer unaware of the experimental group.

### Transmission electron microscopy and quantification of podocyte foot process effacement

Renal cortex tissues were cut into 1 mm cubes and fixed for 4 h at 4°C in 2.5% glutaraldehyde in 0.1mol/L phosphate buffer. They were postfixed in 1% buffered osmium tetroxide, dehydrated through graded ethanols and embedded in Epon. Ultrathin sections (80 nm) were stained and examined by the Hitachi 7500 transmission electron microscope (Hitachi, Tokyo, Japan). The podocyte foot-process effacement was quantified as the method described previously 28.

### Immunohistochemistry

The sections were deparaffinized in xylene, rehydrated in decreasing percentage of ethanol solutions, and incubated in 3% H_2_O_2_ for 20 min at 37 °C. They were then washed three times with PBS for 5 min. Antigen retrieval was performed in a microwave oven, and the sections were then blocked in 5% bovine serum albumin. The sections were incubated overnight with an anti-Flot2 primary antibody (1:100 dilution) at 4°C. Following washing, the sections were incubated with affinity-purified biotinylated goat anti-rabbit antibody or anti-mouse IgG. The protein was visualized using diaminobenzidinetetrahydro-chloride as a chromogen. Tissue sections were counterstained with Mayer's hematoxylin. These stained slides were observed under a microscope and photographed. The distribution and expression of positive protein in rat kidney tissues were detected by measuring the mean value of positive staining area. Data were statistically analyzed using Image-Pro Plus 6.0 software.

### Sucrose density gradient fractionation

The cultured podocytes under different experimental conditions or the grinded kidney tissues of podocyte-specific flot2 knockout or knockin mice with LPS treatment were resuspended in lysis buffer (10mM Tris-HCl (pH 7.4), 1mM EDTA, 1mM EGTA, and 1% Triton X-100) supplemented with 1mM Na3VO4 and 1% protease inhibitor cocktail. After the lysates were placed on ice and homogenized with a Dounce homogenizer, the total volume was adjusted to 2 mL by the addition of lysis buffer. A 2 mL aliquot of 85% sucrose was added to the lysate, and the combination was mixed completely and transferred to a high-speed centrifugation tube. The mixture was then overlaid sequentially with 4 mL of 35% sucrose and 4 mL of 5% sucrose, and the tube was centrifuged at 60000rpm for 20 h at 4°C in a SW41Ti rotor (Beckman, Brea, CA, USA). After centrifugation, 12 fractions of 1ml were then collected sequentially starting from the top of the gradient and analyzed by western blotting.

### Western blotting

Cultured podocytes under different experimental conditions were conducted as previously described 27,29. Twelve protein fractions collected from the sucrose density gradient centrifugation mentioned above were stored at -20 °C and then analyzed by western blotting. The protein concentration was evaluated using a BCA protein assay reagent kit (Invitrogen, Thermo Fisher Scientific, Waltham, MA). Equal amount of proteins was separated on 9% sodium dodecyl sulfate-polyacrylamide gels and transferred onto polyvinylidene fluoride (PVDF) membranes (Millipore, Billerica, MA, USA). The membranes were blocked in 5% nonfat dry milk in TBS-Tween for 1h at RT followed by overnight at 4°C incubation with the following primary antibodies: rabbit polyclonal anti-Flot2 (1:1000, Cell Signaling Technology, USA), rabbit anti-GAPDH (1:5000, Bioworld Technology,Nanjing, China), rabbit anti-nephrin (Abcam), rabbit anti-Podocin (Sigma), anti-CD71(TfR, 1:200, Santa Cruz Biotechnology, Santa Cruz, CA). After washing in TBS-Tween (3 times for 10 min), horseradish peroxidase conjugated goat anti-rabbit or goat anti-mouse IgG at a concentration of 1:3000 was applied for 1h at RT. Bands were visualized using enhanced chemiluminescence (ECL) reagent (Advansta, Menio Park, CA, USA).

### Plasmid constructs

The following plasmids were prepared. Plasmids encoding HA-tagged flot2 domains F1 (amino acids 1-30), F2(amino acids 30-184), F3 (amino acids 184-428), F1+2 (amino acids 1-184), F1+3 (amino acids 1-30+amino acids 184-428) and Full-length flot2 (amino acids 1-428), as well as Flag-tagged podocin domains P1+2 (amino acids 1-128), P3 (amino acids 128-284), P4 (amino acids 284-385), P2+3 (amino acids 107-284), P3+4 (amino acids 128-385) and Full-length podocin (amino acids 1-385) in the pReceiver vector were purchased from Genecopoeia (Guangzhou, China). A mouse Full-length flot2 cDNA (amino acids 1-428) and flot2 cDNA fragment encoding the 155 amino acids (SPFH, amino acids 30-184) flanked with BamHI and XhoI restriction sites were subcloned into pGEX-CBP-GST-MCS vector (GE Healthcare Biosciences, Piscataway, NJ). To express the His-tagged mouse podocin and podocin-SPFH domain, a mouse full-length podocin cDNA and podocin cDNA fragment encoding the 157-amino acid residues (SPFH, amino acids 128-284) flanked with BamHI and XhoI restriction sites were cloned into the pET-28a(+) vector (Fitgene Technology Co., Ltd, Guangzhou, China). Restriction digestion and DNA sequencing were performed to validate all constructs.

### Co-immunoprecipitation (Co-IP)

HA-tagged Full-length flot2 (amino acids 1-428) and flot2 domains F1(amino acids 1-30), F2(amino acids 30-184), F3 (amino acids 184-428), F1+2 (amino acids 1-184), F1+3(1-30 amino acids + amino acids 184-428), as well as Flag tagged- Full-length podocin (amino acids 1-385) and domains P1+2 (amino acids 1-128), P3 (amino acids 128-284), P4 (amino acids 284-385), P2+3 (amino acids 107-284), P3+4 (amino acids 128-385) were expressed in HEK-293T cells. Experiments on endogenous proteins were carried out in differentiated podocytes and renal cortex from *C57Bl/6* mice, respectively. Renal cortex from *C57Bl/6* mice was homogenized on ice in hypotonic buffer with 20 strokes of a hand-driven glass homogenizer. Cells and renal cortex pellets were resuspended in lysis buffer with protease inhibitor mixtures (Roche, Indianapolis, IN).The samples were centrifuged and the supernatant were incubated with primary antibodies anti-Flag (Sigma-Aldrich, St. Louis, Missouri, USA), anti-HA (Invitrogen, Thermo Fisher Scientific, Waltham, MA), anti-podocin (Sigma-Aldrich, St. Louis, Missouri, USA), anti-flot2 (Santa Cruz Biotechnology, Santa Cruz, CA), or IgG for 4 h at 4 °C, followed by the addition of 20 μl of protein A/G agarose (Invitrogen, Thermo Fisher Scientific, Waltham, MA) for 12 h. Co-immunoprecipitation were performed using the Pierce Co-Immunoprecipitation Kit (26149, Pierce; Rockford, IL) per the manufacturer's instructions.

### Pull down assay

Plasmids encoding glutathione S-transferase (GST) and Histidine (His) were generated by cloning restriction-digested PCR products into the pGEX-CBP-MCS and pET-28a(+) expression vector (Fitgene Technology Co., Ltd, Guangzhou,China).The fidelity of all DNA constructs was confirmed by DNA sequencing. For pull-down experiments, the GST-flot2 (full length or amino acids 30-184) fusion proteins, and His-podocin fusion proteins (full length or amino acids 128-284) were respectively extracted from the Escherichia coli Bl21 (Stratagene, La Jolla, California, USA). Bacterial cells were cultured in LB medium containing ampicilin until the OD600 reaches 0.6-0.8, followed by adding 100 mM IPTG to induce recombinant protein expression. After shaking at room temperature for 3 h, cell pellets were collected by spinning at 5,000g for 10 min at 4 °C. GST resin (#17-0756-01,GE Healthcare Biosciences, Piscataway, NJ) or His resin (#17-5318-01,GE Healthcare Biosciences, Piscataway, NJ) were used to incubate with bacterial cell lysis at 4 °C for 1h. GST or His Resin were washed three times with lysis buffer, and then respectively incubated with His Resin or GST resin overnight at 4 °C. GST or His resin was respectively eluted with 20 mM reduced glutathione (G4251-5g, Sigma-Aldrich, St. Louis, Missouri, USA) and with 200mM imidazole (#18005, Fitgene Technology Co., Ltd, Guangzhou, China) for 10min. Eluate were eluted into SDS sample buffer by heating to 100°C for 10 min. The entire sample was loaded onto 10% SDS-PAGE gels and then analyzed by immunoblotting with anti His (#CW0286A, CoWin Biosciences, Guangzhou, China) or anti GST (#HT601, TransGen Biotech, Beijing, China).

### ChIP-quantitative PCR assay

ChIP assay was done as described 27, 29 using the Thermo ChIP Kit (Invitrogen). Cells (2x10^6^) were crosslinked with a 1% formaldehyde solution for 10 min and terminated by adding lycine 1.25 M). After being washed twice using ice-cold PBS, cells were harvested in cell scraper and then lysed in 200 μL of SDS lysis buffer, and sonicated to generate 300 to 800 bp DNA fragments. The lysate was pre-cleared by incubation with protein G agarose and incubated overnight at 4 °C with either anti-KLF15 antibody (Santa Cruz Biotechnology) or non-immune IgG (Upstate Biotechnology, Inc.). To collect the immunoprecipitated complexes, protein G magnetic beads were incubated. After purified, DNA samples were amplified in a 7500 quantitative real-time PCR System (Applied Biosystems, Carlsbad, CA) and quantitated in triplicate by SYBR Green qPCR (Takara Biotechnology, Dalian, China) using forward and reverse primer sequences (Supplementary Table S3) for the mouse flot2 promoter. Data were analyzed using the 2^-△△^CT method.

### Transfection and luciferase reporter assays

Lentiviral packaging GLuc-ON promoter reporter clone Flot2 plasmids (pEZX-Lv PG04-flot2, GeneCopoeia, Guangzhou, China) were generated in HEK293T cells that were expanded and transfected using the Lenti-Pac HIV Expression Packing Kit (HPK-LvTR-20, GeneCopoeia, Guangzhou).The cell culture medium was collected under different conditions, and luciferase activity was measured using the Secrete-Pair Dual Luminescence Assay Kit (SPDAD010, GeneCopoeia, Guangzhou, China) according to the manufacturer's instructions. Each transfection was repeated in triplicate.

### Statistical analysis

Statistical analyses were performed using SPSS (version 20.0; SPSS Inc., Chicago, IL, USA), GraphPad Prism (version 5.0; GraphPad Software, Inc., La Jolla, CA, USA). Data are expressed as means ± S.E. Student's t-test was employed for comparisons between two groups; one-way analysis of variance (ANOVA) followed by Tukey's post-test for multiple comparisons was used for groups of three or more. The Spearman's test was used for the correlation between flot2 protein expression with eGFR and proteinuria. All tests were two-tailed, and *P*< 0.05 was considered statistically significant.

## Results

### Flot2 was reduced in injured podocytes *in vivo* and *in vitro*

**Figure 1:**
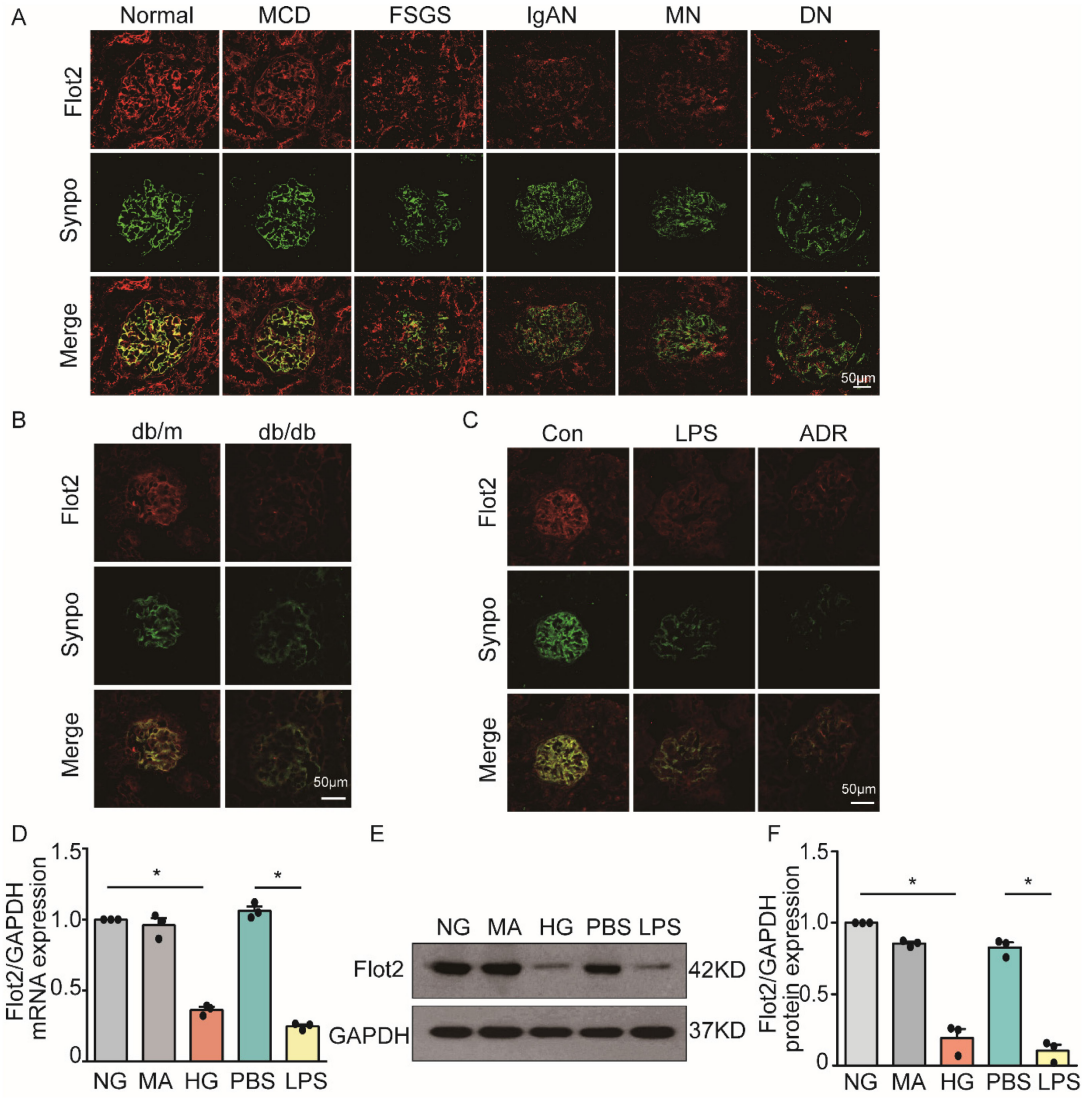
** Flot2 was reduced in podocytes *in vivo* and *in vitro*. (A)** Flot2 expression in podocytes in proteinuric patients. Flot2 protein(red) in podocytes shown by double immunofluorescence using the podocyte marker synaptopodin (synpo, green), resulting in partial yellow overlap. Fot2 was expressed in human glomerular cells, including podocytes, in contrast, podocyte Flot2 expression was significantly reduced in glomeruli from proteinuric patients with minimal change disease (MCD, n=6), focal segmental glomerulosclerosis (FSGS, n=7), IgA nephropathy (IgAN, n=4), membranous nephropathy (MN, n=8), and diabetic nephropathy (DN, n=5). **(B-C)** Flot2 expression in podocytes in murine models of proteinuria. Compared with control mice (*C57BL/6* or *BKS*), Flot2 expression was decreased in murine models of diabetic nephropathy (db/db mice) (n=6) and proteinuria (LPS- or ADR-treated *C57BL/6*, n=6). **(D-F)** Flot2 mRNA and protein levels were decreased in podocytes treated with HG or LPS for 48 h (n=4). Data from at least three independent experiments. ^*^P<0.05 versus controls.

Flot2 is a ubiquitously expressed and highly conserved raft protein. We examined the expression of Flot2 in normal renal tissues from subjects with renal carcinoma (n=3) and human glomerular diseases by immunofluorescence staining of renal biopsy samples from patients with proteinuric glomerular diseases, including minimal change disease (MCD, n=6), focal segmental glomerulosclerosis (FSGS, n=7), IgA nephropathy (IgAN, n=4), membranous nephropathy (MN, n=8), and diabetic nephropathy (DN, n=5). We found Fot2 was expressed in human glomerular cells (Figure 1A), including podocytes, which were identified by podocyte specific marker synaptopodin labeling. In contrast, podocyte Flot2 expression was significantly reduced in glomeruli from proteinuric patients with MCD, FSGS, IgAN, MN, and DN. Flot2 expression in the kidney was also reduced in diabetic db/db mice (Figure 1B), and in lipopolysaccharide (LPS)- induced and adriamycin (ADR)-induced nephropathy mice, as shown by double immunostaining of Flot2 and synaptopodin (Figure 1C). *In vitro*, high glucose (HG) or LPS significantly reduced podocyte Flot2 expression (Figure 1D-F). These results indicated that Flot2 was reduced in injured podocytes *in vivo* and *in vitro*.

### Glomerular Flot2 protein expression was positively correlated with estimated glomerular filtration rate (eGFR) and negatively correlated with proteinuria in proteinuric glomerular diseases

We further assessed the expression of Flot2 in renal biopsies from patients with MCD (n=7), FSGS (n=8), MN (n=8), IgAN (n=5), and DN (n=6) compared with normal renal tissues from subjects with renal carcinoma (n=3) by immunohistochemistry (IHC) (Supplementary Figure S2). The clinical data for these patients are shown in Supplementary Table S4. Flot2 protein expression was markedly decreased in renal biopsies from patients with different forms of proteinuric glomerular diseasesaccording to IHC (Supplementary Figure S2A-B). Furthermore, Spearman's correlation analysis demonstrated that Flot2 protein levels were negatively correlated with proteinuria (Supplementary Figure S2C) and positively correlated with eGFR (Supplementary Figure S2D) in all subjects. These results indicate that Flot2 may contribute to kidney protection.

### Podocyte-specific Flot2 deletion exacerbated albuminuria and kidney injury in LPS- and ADR-induced nephropathy mice

**Figure 2:**
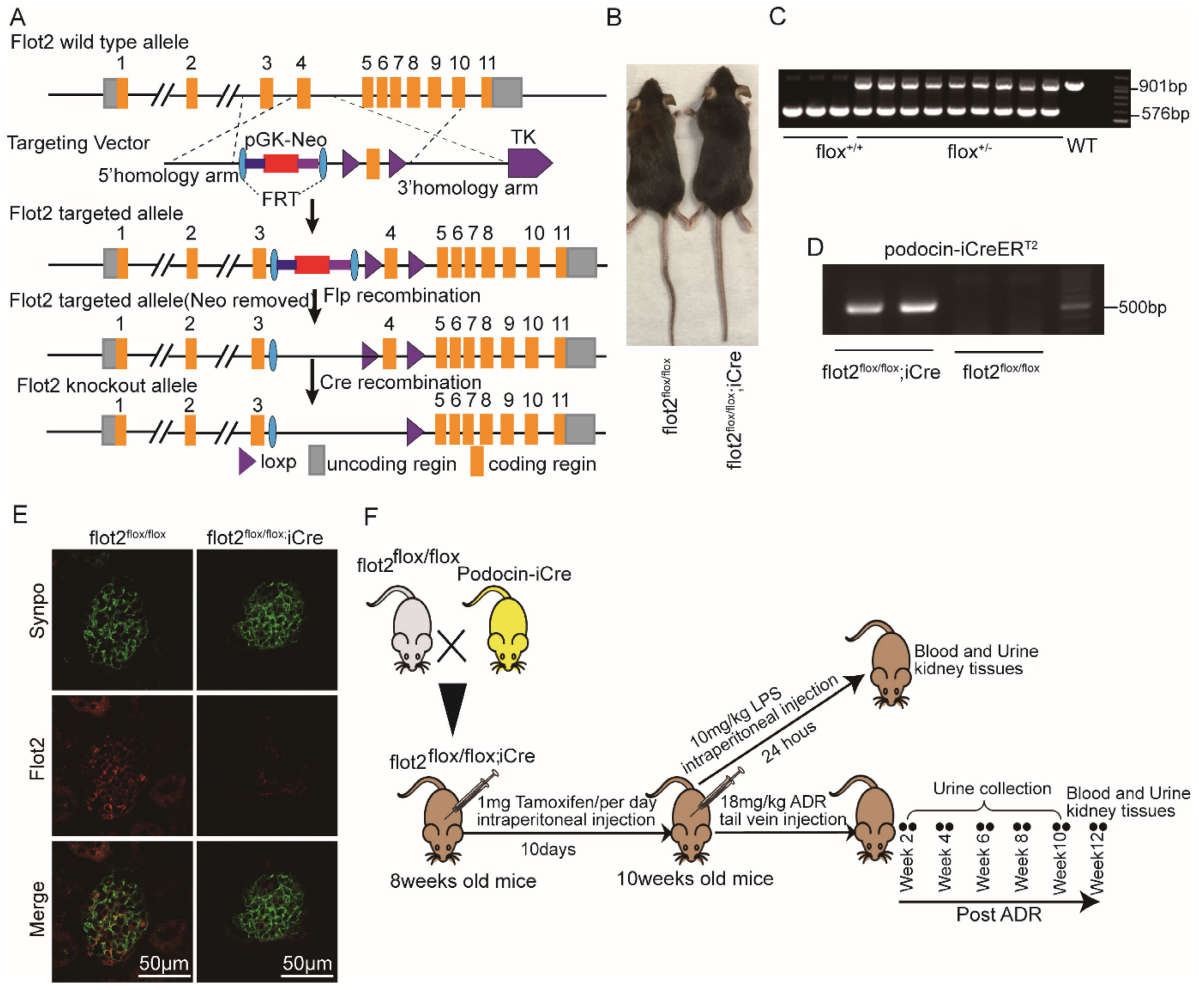
** Generation of conditional podocyte-specific flot2 knockout mice. (A)** constructive strategy of flot2 gene knockout by using Cre-LoxP recombination system. Exon 4 is deleted upon podocin-iCre mediated recombination. **(B)** macroscopic characteristics of flot2^flox/flox^ and flot2^flox/flox;iCre^mice. **(C)** PCR analysis authenticated flot2 after homologous recombination of loxP-stop-loxP-flot2. **(D)** PCR analysis authenticated iCre after homologous recombination of podocin-iCreERT2. **(E)** representative double-immunofluorescence of flot2 and Synpo in glomerular podocytes. Flot2, red; Synpo, green; Merge, yellow. Scale bar: 50 μm.** (F)** A schematic diagram showing the method of building LPS or ADR-induced nephropathy model. Eight weeks old flot2^flox/flox^ mice were given intraperitoneal injection of 200μg LPS or caudal vein injection ADR (18mg/kg) and sacrificed after 24 hours or 12 weeks.

**Figure 3:**
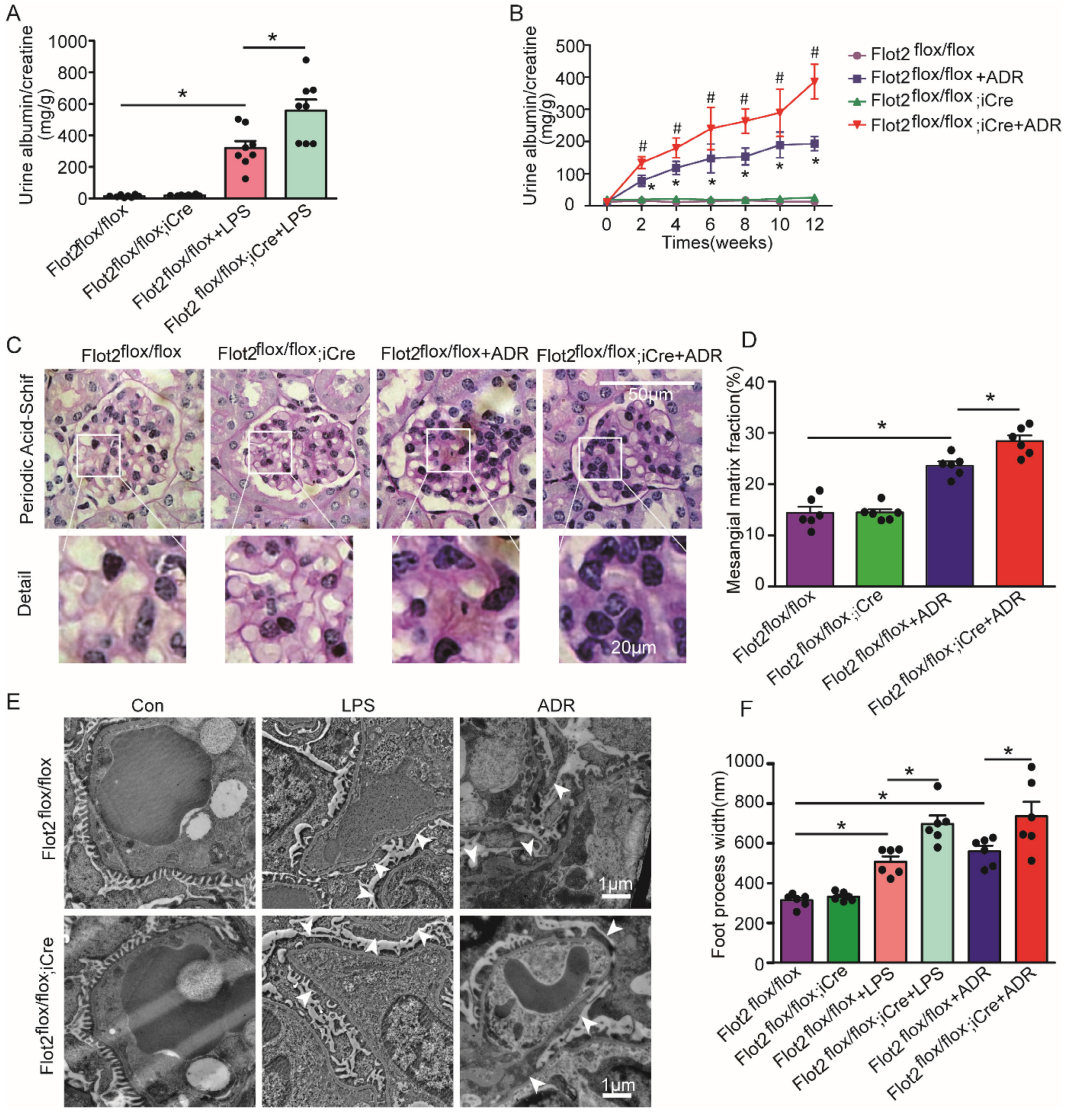
** Podocyte-specific *Flot2* deletion exacerbated albuminuria and kidney injury in LPS- and ADR-induced nephropathy mice. (A)** Urinary albumin excretion (mg/g creatinine) in Podocin-iCre Flot2^flox/flox^ mice and control littermates injected with 200μg LPS at 24 h. **(B)** Urinary albumin excretion (mg/g creatinine) in Podocin-iCre Flot2^flox/flox^ mice and control littermates injected with ADR (18 mg/kg) via caudal vein at the indicated times. **(C-D)** Representative periodic acid-Schiff staining for glomerular mesangial matrix at age 12 weeks (n=6). Scale bar, 50 μm. Semiquantitative sclerosis analysis of mesangial matrix expansion by mesangial matrix fraction (%) (n=50 glomeruli per group).** (E-F)** Representative electron microscopy observation of podocyte foot process effacement. Scale bar, 1 μm. Quantification of podocyte foot process effacement by foot process width (nm). Data shown as mean ± standard error. ^*^P<0.05 versus Flot2^flox/flox^; ^#^*P*<0.05 versus Flot2^flox/flox^+LPS or Flot2^flox/flox^ +ADR.

We assessed the effects of reduced Flot2 expression in proteinuria and kidney injury in C57BL/6 mice with podocyte-specific targeted deletion of the Flot2 gene and in proteinuric model mice induced by LPS and ADR (Figure 2F). Compared with LPS-treated control mice, Podocin-iCre Flot2^flox/flox^mice had significantly higher urine albumin levels 24 h after LPS injection (Figure 3A), indicating that podocyte-specific Flot2 deletion exacerbated albuminuria. Similarly, ADR-treated Podocin- iCre Flot2^flox/flox^ mice exhibited a significant increase in albuminuria at around 2 weeks after ADR injection compared with all the other groups. Albuminuria increased in ADR-treated Podocin-iCre Flot2^flox/flox^ mice in a time-dependent manner (Figure 3B). Periodic acid-Schiff staining revealed increased mesangial expansion in ADR-treated Podocin-iCre Flot2^flox/flox^ mice compared with the other groups (Figure 3C-D). Transmission electron microscopy showed podocyte foot process effacement and width were markedly increased in podocyte *Flot2*-deletion mice treated with LPS or ADR compared with all other groups (Figure 3E-F), supporting the idea that Flot2 deletion exacerbated podocyte injury. Overall, these results suggest that knockout of Flot*2* in podocytes increased the susceptibility to LPS- and ADR-induced podocyte and kidney injury.

### Podocyte-specific Flot2 overexpression ameliorated albuminuria and kidney injury in LPS- and ADR-induced nephropathy mice

**Figure 4:**
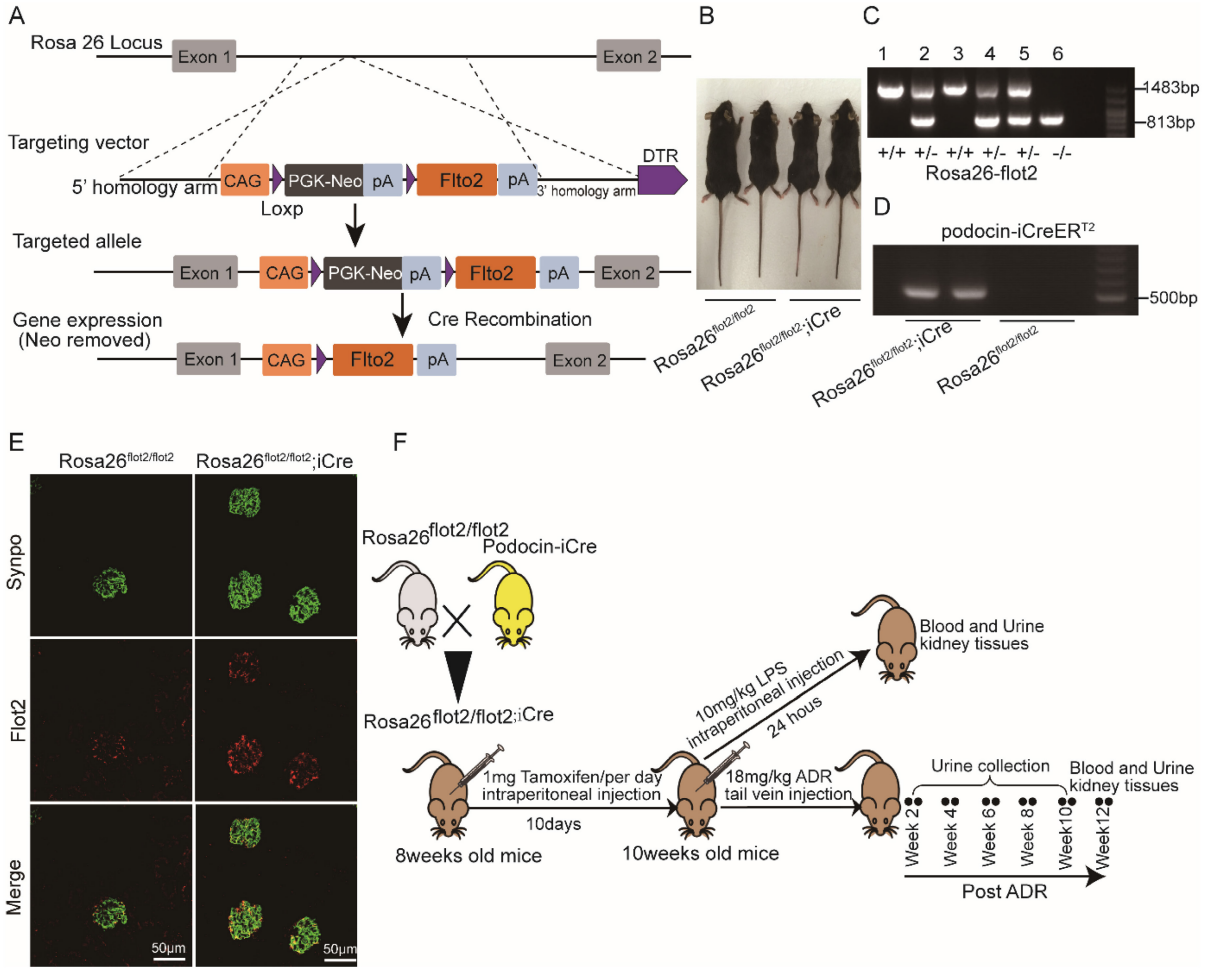
** Generation of conditional podocyte-specific flot2 knockin mice. (A)** constructive strategy of flot2 gene knockin illustrated the Rosa26 targeting loci, flot2 targeting vector structure, and the conditional allele after homologous recombination. **(B)** Macroscopic characteristics of Rosa26^flot2/flot2^ and Rosa26^flot2/flot2;iCre^ mice.** (C)** PCRanalysis authenticated Rosa26*-*flot2 after homologous recombination of loxP-stop-loxP-flot2.** (D)** PCR analysis authenticated iCre after homologous recombination of podocin-iCreERT2.** (E)** Representative double-immunofluorescence of flot2 and Synpo in glomerular podocytes. Flot2, red; Synpo, green; Merge, yellow. Scale bar: 50 μm.** (F)** A schematic diagram showing the method of building LPS or ADR-induced nephropathy model. Eight weeks old Rosa26^flot2/flot2;iCre^ mice were given intraperitoneal injection of 200μg LPS or caudal vein injection ADR (18mg/kg) and sacrificed after 24 hours or 12 weeks.

**Figure 5:**
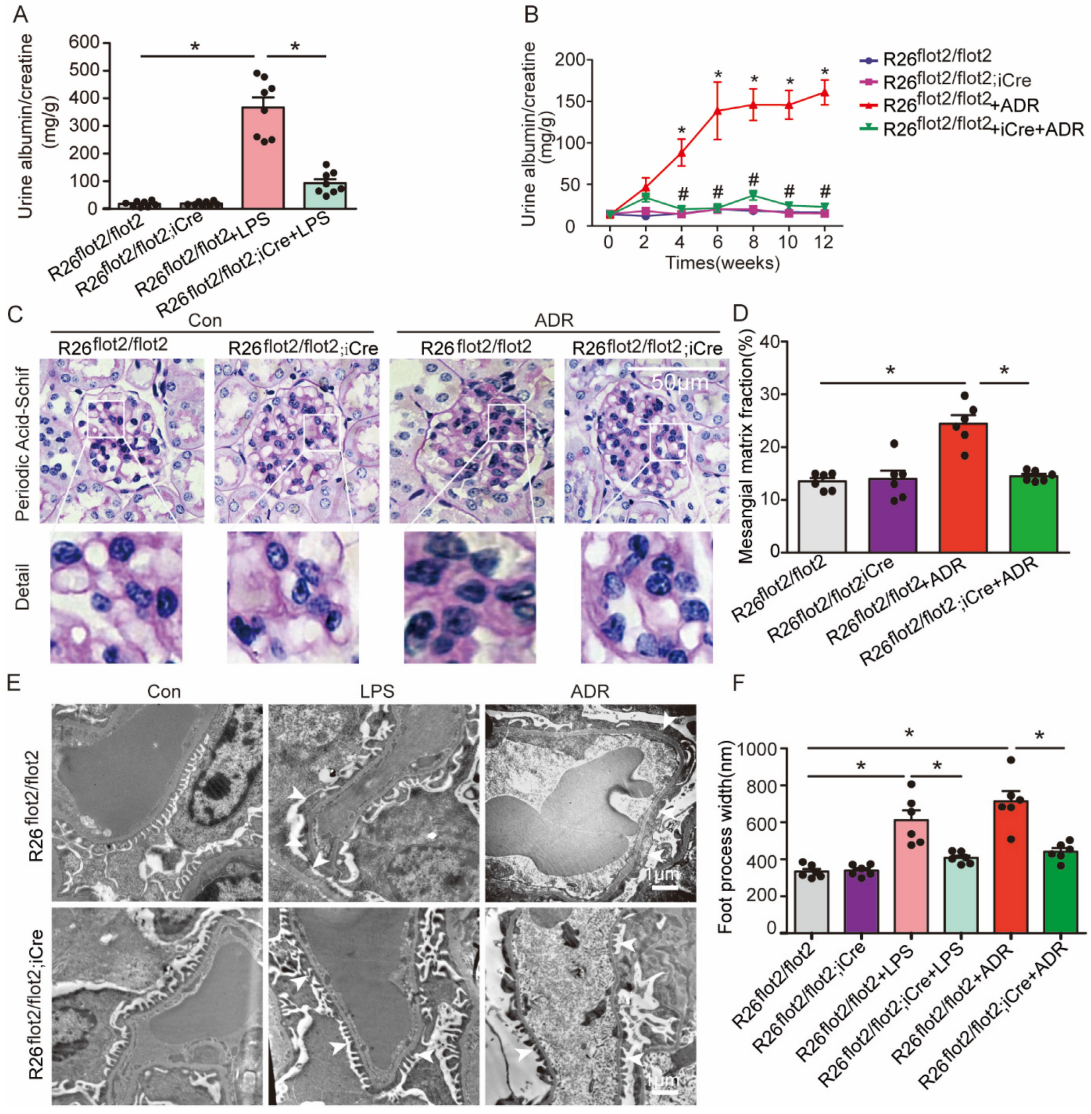
** Podocyte-specific *Flot2* overexpression ameliorated albuminuria and kidney injury in LPS- and ADR-induced nephropathy mice. (A)** Urinary albumin excretion (mg/g creatinine) in R26^flot2/flot2^Podocin-iCre mice treated with LPS. **(B)** Urinary albumin excretion (mg/g creatinine) in ADR-treated R26^flot2/flot2^ Podocin-iCre mice after 12 weeks. **(C-D)** Representative periodic acid-Schiff staining for glomerular mesangial matrix at age 12 weeks. Scale bar, 50μm. Semiquantitative sclerosis analysis of mesangial matrix expansion by mesangial matrix fraction (%) (n=50 glomeruli per group). **(E-F)** Representative electron microscopy observation of podocyte foot process effacement. Scale bar, 1 μm. Quantification of podocyte foot process effacement by foot process width (nm). Data shown as mean ± standard error.^*^P<0.05 versus R26^flot2/flot2^; ^#^*P*<0.05 versus R26^flot2/flot2^+LPS or R26^flot2/flot2^+ ADR.

We further examined the podocyte-protective role of Flot2 *in vivo* by generating and validating R26^flot2/flot2^ Podocin-iCre mice on a *C57BL/6* background (Figure 4), and using mouse models of podocyte injury and albuminuria induced by intraperitoneal LPS or tail-vein ADR injection (Figure 4). Compared with LPS-treated control mice, R26^flot2/flot2^ Podocin-iCre mice treated with LPS exhibited significantly less albuminuria 24 h after LPS injection (Figure 5A), indicating that Flot2 overexpression in podocytes was sufficient to reduce albuminuria. Similarly, R26^flot2/flot2^ Podocin-iCre ADR-induced nephropathy model mice also exhibited a significant decrease in albuminuria at 4-12 weeks after ADR injection compared with all other groups (Figure 5B). In addition, mesangial matrix expansion was attenuated in ADR-treated R26^flot2/flot2^ Podocin-iCre mice compared with their control littermates treated with ADR (Figure 5C-D). Transmission electron microscopy showed podocyte foot-process fusion in LPS-treated control mice, and this change was ameliorated by podocyte Flot2 knockin (Figure 5E-F), suggesting that podocyte Flot2 transgene expression alleviated podocyte injury. Similarly, electron microscopy also showed that foot process effacement and width were significantly attenuated in R26^flot2/flot2^ Podocin-iCre mice compared with their control littermates at 12 weeks after ADR injection (Figure 5E-F). Collectively, these data suggest that podocytes contribute to the protective effects of Flot2 overexpression against LPS- and ADR-induced injury.

### Flot2 governs the arrangement of the actin cytoskeleton in podocytes *in vitro*

**Figure 6:**
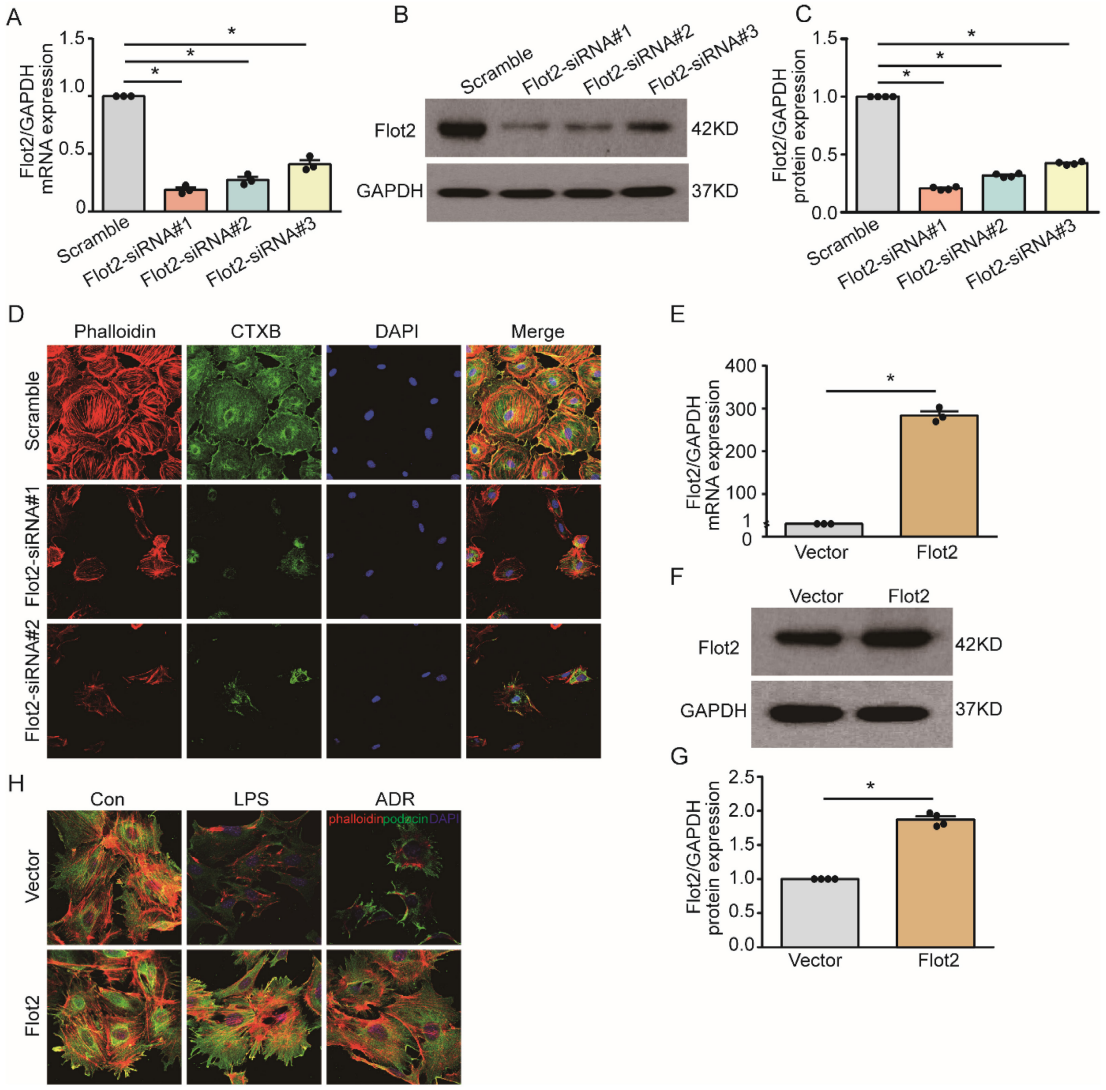
** Flot2 governs the arrangement of the actin cytoskeleton in podocytes *in vitro*. (A-C)** Flot2 was silenced using Flot2-siRNA and knockdown of Flot2 mRNA and protein expression levels were confirmed by quantitative real-time RT-PCR analysis and immunoblotting, respectively.** (D)** Phalloidin labeling of actin filaments (red) and cholera toxin B pentamer (CTxB) labeling of raft (green) were used to probe actin cytoskeletal rearrangements and raft distribution. Nuclei were stained with 4',6-diamidino-2-phenylindole. Flot2 knockdown resulted in reorganization of the actin filaments and dissolution of the cytoplasmic radial stress fibers, and stimulated cell contraction, resulting in smaller cell size. **(E-G)** A cell model of Flot2 overexpression in podocytes was established. Flot2 mRNA and protein expression were increased in podocytes treated with adenovirus containing GFP-Flot2. Data from at least three independent experiments.^ *^P<0.05 versus vector.** (H)** Cytoskeleton disorders were detected in LPS- and ADR-injured podocytes labelled with phalloidin (red) and podocyte marker podocin (green) using double immunofluorescence.LPS- and ADR-induced actin rearrangements and cell contraction were abolished by transfection with adenovirus containing Flot2.

Cytoskeletal reorganization plays an important pathogenic role in podocyte injury and proteinuria. We explored the effect of Flot2 on cytoskeletal structure in podocytes *in vitro* using a cell model of Flot2-knockdown using small interfering RNA (siRNA). Western blotting and quantitative real-time reverse transcription-polymerase chain reaction (RT-PCR) confirmed efficient siRNA- mediated Flot2 gene silencing in podocytes (Figure 6A-C). We then probed actin cytoskeletal rearrangement and raft distribution using phalloidin labeling of actin filaments and cholera toxin B pentamer (CTxB) labeling, respectively, and examined the functional relevance of Flot2 knockdown in podocytes. Flot2 knockdown resulted in reorganization of actin filaments and dissolution of cytoplasmic radial stress fibers (Figure 6D), and stimulated cell contraction, resulting in smaller cell size. We confirmed the role of Flot2 in podocyte cytoskeletal structure by establishing a cell model of Flot2-overexpressing podocytes using adenovirus containing Flot2. Adenovirus containing Flot2 markedly increased Flot2 mRNA and protein levels in treated podocytes (Figure 6E-G). We also investigated the effect of overexpression of Flot2 on cytoskeleton disorders in podocytes induced by LPS or ADR. LPS- and ADR-induced actin rearrangement and cell contraction was abolished by transfection with adenovirus containing Flot2 (Figure 6H), supporting the notion that Flot2 causes rearrangement of the actin cytoskeleton in podocytes.

### Location of SD proteins in lipid rafts relied on Flot2

**Figure 7:**
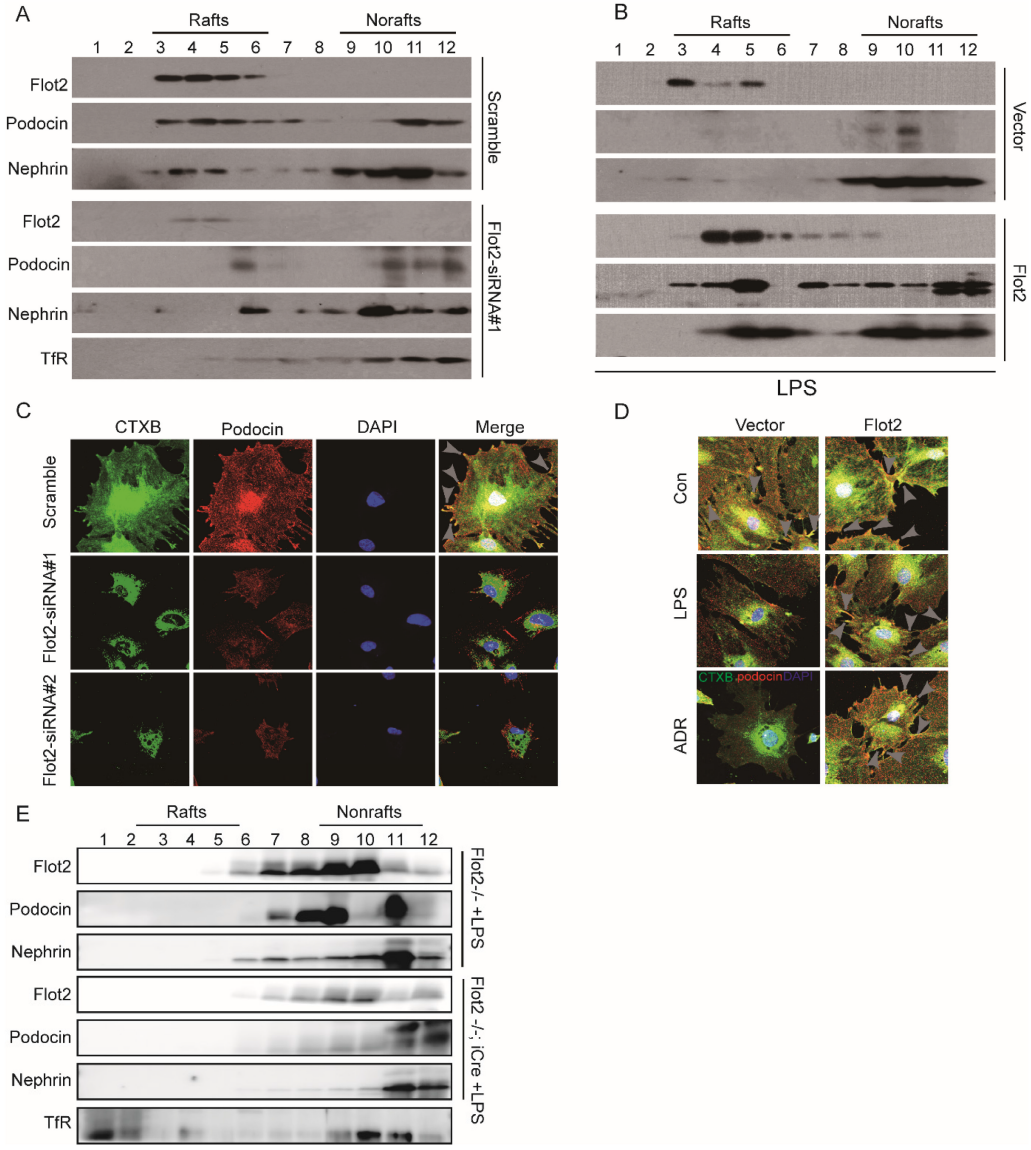
** Location of slit membrane proteins in lipid rafts relied on Flot2.** Cellular lysates under different conditions were subjected to sucrose density-gradient centrifugation. Twelve fractions were collected starting from the surface of the centrifuged liquid, and were analyzed by western blot with respective antibodies. The non-raft plasma membrane protein transferrin receptor served as a control to indicate the purity of the preparation. **(A)** Flotation analysis of the slit diaphragm (SD) proteins podocin and nephrin in podocytes after Flot2 knockdown. The plasma membrane distributions of Flot2, podocin and nephrin were significantly reduced in lipid rafts in podocytes following Flot2-siRNA knockdown compared with cells treated with scrambled control siRNA. **(B)** Flotation analysis of Flot2, podocin and nephrin in LPS-treated podocytes with and without Flot2 overexpression. SD protein expression levels were strongly reduced in lipid rafts in LPS-treated podocytes, while podocin and nephrin were detected in lipid rafts in LPS-treated podocytes with Flot2 overexpression.** (C)** CTxB labeling raft (green) was used to probe the distribution of podocin (red) in rafts in Flot2-siRNA knockdown podocytes, resulting in yellow overlap. Nuclei were stained with 4',6-diamidino-2-phenylindole. Flot2-siRNA knockdown reduced podocin distribution in lipid rafts in podocytes. **(D)** CTxB labeling of rafts was used to probe the distribution of podocin in rafts in LPS- and ADR-treated podocytes with and without Flot2 overexpression. Podocin distribution in lipid rafts was reduced in LPS- and ADR-treated podocytes, while overexpression of Flot2 in podocytes restored podocin expression in lipid rafts. **(E)** Distribution of SD proteins podocin and nephrin in podocyte-specific Flot2-deletion mice treated with LPS.The reductions in podocin and nephrin in lipid rafts were exacerbated in LPS-treated Podocin-iCre Flot2^flox/flox^ mice.

SD proteins regulate podocyte cytoskeletal dynamics, protein expression, and cell survival 4, 5,14. Lipid raft microdomains are critical for the dynamic functional organization of the SD in podocytes 9. We examined the effect of the raft protein Flot2 on SD proteins by observing the location of the SD proteins podocin and nephrin in lipid rafts in Flot2-knockdown and Flot2-overexpressing podocytes, transiently transfected with Flot2-siRNA or adenovirus containing Flot2, respectively. Cellular lysates were subjected to sucrose density-gradient centrifugation and 12 fractions were collected, starting from the surface of the centrifuged liquid. The non-raft plasma membrane protein transferrin receptor was excluded, while Flot2 and the SD proteins podocin and nephrin were highly enriched in control cells (Figure 7A). However, Flot2 knockdown reduced the plasma membrane distribution of these SD proteins in lipid rafts. Similar results were observed in LPS-treated podocytes. In contrast, podocin and nephrin were detected in lipid rafts in the presence of Flot2, suggesting a role for Flot2 in recruiting these SD proteins in lipid rafts (Figure 7B).

We also investigated podocin distribution in the rafts using CTxB labeling, and examined the functional relevance of Flot2 knockdown in podocytes *in vitro*. Flot2-siRNA knockdown reduced podocin distribution in lipid rafts, and similar results were obtained in LPS- and ADR-treated podocytes (Figure 7C). In contrast, overexpression of Flot2 in podocytes restored podocin expression in lipid rafts (Figure 7D). We confirmed the role of Flot2 in trafficking SD proteins in lipid rafts in podocytes *in vitro* by investigating the distributions of podocin and nephrin in podocyte-specific Flot2*-*deletion mice treated with LPS. The reductions in podocin and nephrin in lipid rafts were exacerbated in LPS-treated Podocin-iCre Flot2^flox/flox^ mice compared with control mice that received LPS (Figure 7E). This data confirmed our *in vitro* results and further indicated that Flot2 may recruit SD proteins into lipid rafts.

### Flot2 directly interacts with podocin each other via their SPFH domain

**Figure 8:**
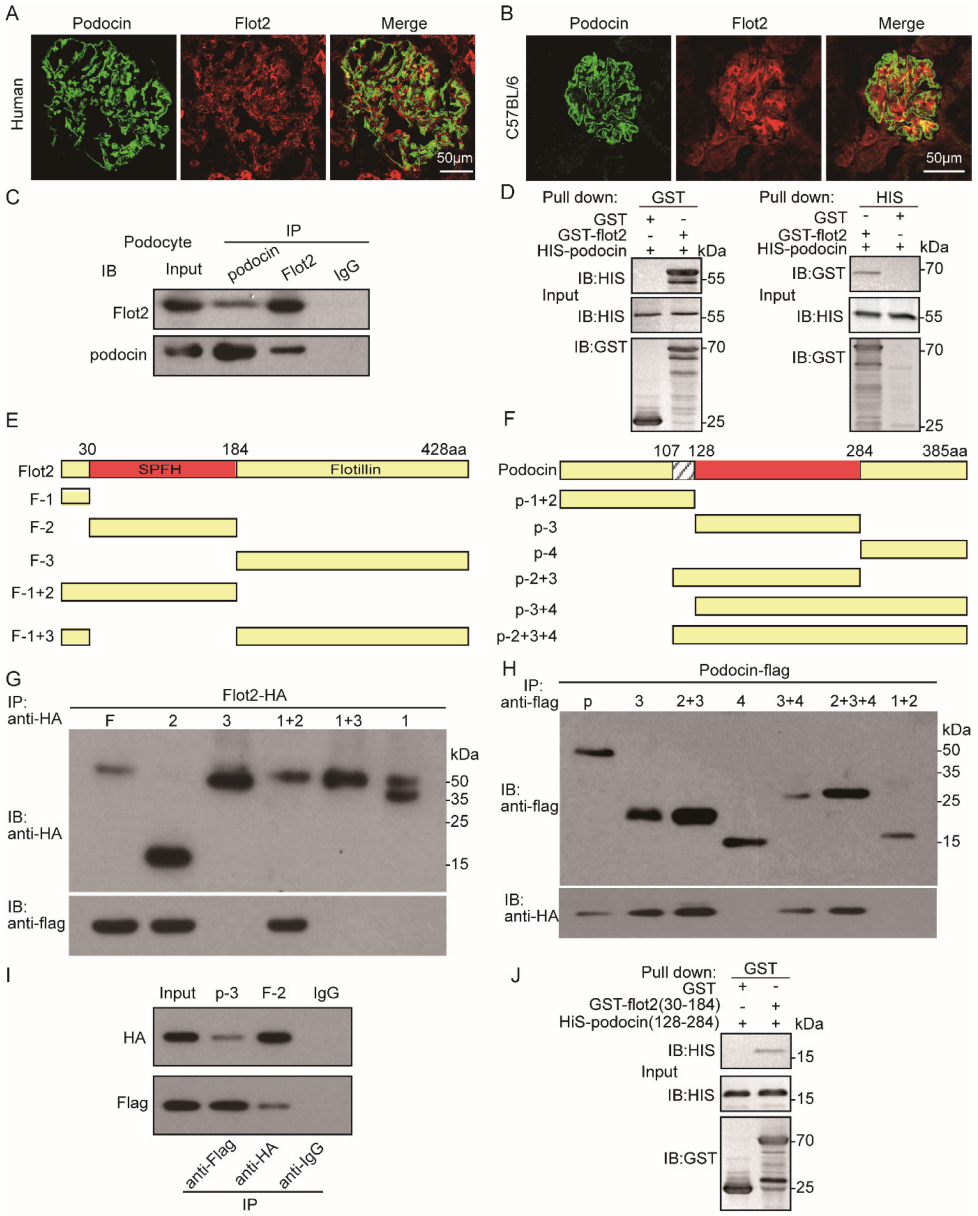
** Flot2 directly interacts with podocin each other via their SPFH domain. (A-B)** Partial colocalization of Flot2 and podocin was ascertained by double-label confocal immunofluorescence microscopy in glomerular podocytes of renal cortex from human (adjacent normal renal tissue of renal tumor) and *C57BL* mice. Confocal signal from podocin (green fluorescence) and Flot2(red fluorescence) and a merged signal (yellow). Scale bar: 50μm. **(C)** Coimmunoprecipitation of endogenously expressed proteins in differentiated podocytes. Flot2 interacts with podocin protein *in vitro* cultured podocytes.** (D)** The direct interactions of Flot2 and podocin were confirmed by His- and GST-pull down assays. **(E)** Schematic diagram of the domain structure of Flot2.Five truncations of Flot2 protein were generated. **(F)** Schematic diagram of the domain structure of podocin.Six truncations of podocin protein were generated. **(G-I)** Reciprocal coimmunoprecipitation assay using HA-tagged Flot2 domain constructs and flag-tagged podocin domain constructs were carried out with an antibody against the HA tags on the Flot2 proteins, or Flag tags on the podocin, as indicated. Flot2-HA (F, amino acid 1-428; F2, amino acid 30-184; F1+2, amino acid 1-184,) interactions with podocin could be detected when the initial precipitation was carried out with an antibody against the HA tags on Flot2**(G)**. Conversely, podocin-flag (p, amino acid 1-385;p3, amino acid 128-284; p2+3, amino acid 107-284;p3+4, amino acid 128-385;p2+3+4, amino acid 107-385) interactions with flot2 could be detected when the initial precipitation was carried out with an antibody against the flag tags on podocin**(H)**.**(I)** coimmunoprecipitation carried out in HEK293T cells transiently expressing HA tagged Flot2 truncations (F2, 30-184) and flag tagged podocin truncations (p3, amino acid 128-284). The interaction could be detected regardless of which antibody was used for the initial immunoprecipitation. No signals were detected when immunoprecipitation was carried out with IgG. **(J)** the binding of GST-Flot2 (30-184) to His-podocin (128-284) was further confirmed by GST pull-down assay.

Podocin and Flot2 channels showed a partially overlapping distribution in glomerular podocytes in the renal cortex in humans and C57BL mice, as ascertained by double-label confocal immunofluorescence microscopy (Figure 8A-B). The interaction between podocin and Flot2 in podocytes was examined by co-immunoprecipitation using antibodies against Flot2 and podocin, and an interaction was detected following initial immunoprecipitation with either anti-Flot2 or anti-podocin antibodies, but not following immunoprecipitation with an IgG antibody (Figure 8C).We validated the results of the endogenous co-immunoprecipitation experiments by examining possible direct interactions between Flot2 and podocin by His- and glutathione-S-transferase (GST)-pull-down assays. Bacterially expressed His-tagged podocin and GST-tagged Flot2 or GST recombinant proteins were affinity purified, and protein-protein interactions were examined by GST and His pull-down assays, followed by immunoblotting with anti-His and GST antibodies. GST pull-down assay confirmed the interaction between Flot2 and podocin by western blotting with anti-His antibody (Figure 8D). Similarly, His pull-down assay also confirmed the results by western blotting with anti-GST antibody. Overall, these results indicated that Flot2 interacted directly with podocin in podocytes.

We clarified the protein domains responsible for the specific interaction between Flot2 and podocin by generating five truncated Flot2 and six truncated podocin proteins (Figure 8E-F). We then performed reciprocal co-immunoprecipitation assays using hemagglutinin (HA)-tagged podocin domain constructs and FLAG-tagged podocin domain constructs, respectively. Immunoprecipitation of HEK293T cells lysates with anti-HA antibodies followed by immunoblotting for FLAG revealed that Flot2-HA (amino acids 30-184) co-immunoprecipitated with podocin (Figure 8G). Conversely, podocin-FLAG (amino acids 128-284) co-immunoprecipitated with Flot2 (Figure 8H). This interaction was also observed in HEK-293T cells transiently co-expressing HA-tagged podocin (amino acids 128-284) together with HA-tagged Flot2 (amino acids 30-184) (Figure 8I). The interaction could be detected regardless of which antibody was used for the initial immunoprecipitation. Binding of GST-Flot2 (amino acids 30-184) to His-podocin (amino acids 128-284) was also confirmed by GST pull-down assay. GST-Flot2 (amino acids 30-184) fusion protein was able to pull His-podocin (amino acids 128-284) out of the lysates, whereas GST was ineffective (Figure 8J). These experiments confirmed that amino acids 30-184 of Flot2 formed a complex with amino acids 128-284 of podocin in podocytes.

### Identification of KLF15 as potential upstream transcription factor mediating Flot2 transcriptional activity and expression

**Figure 9:**
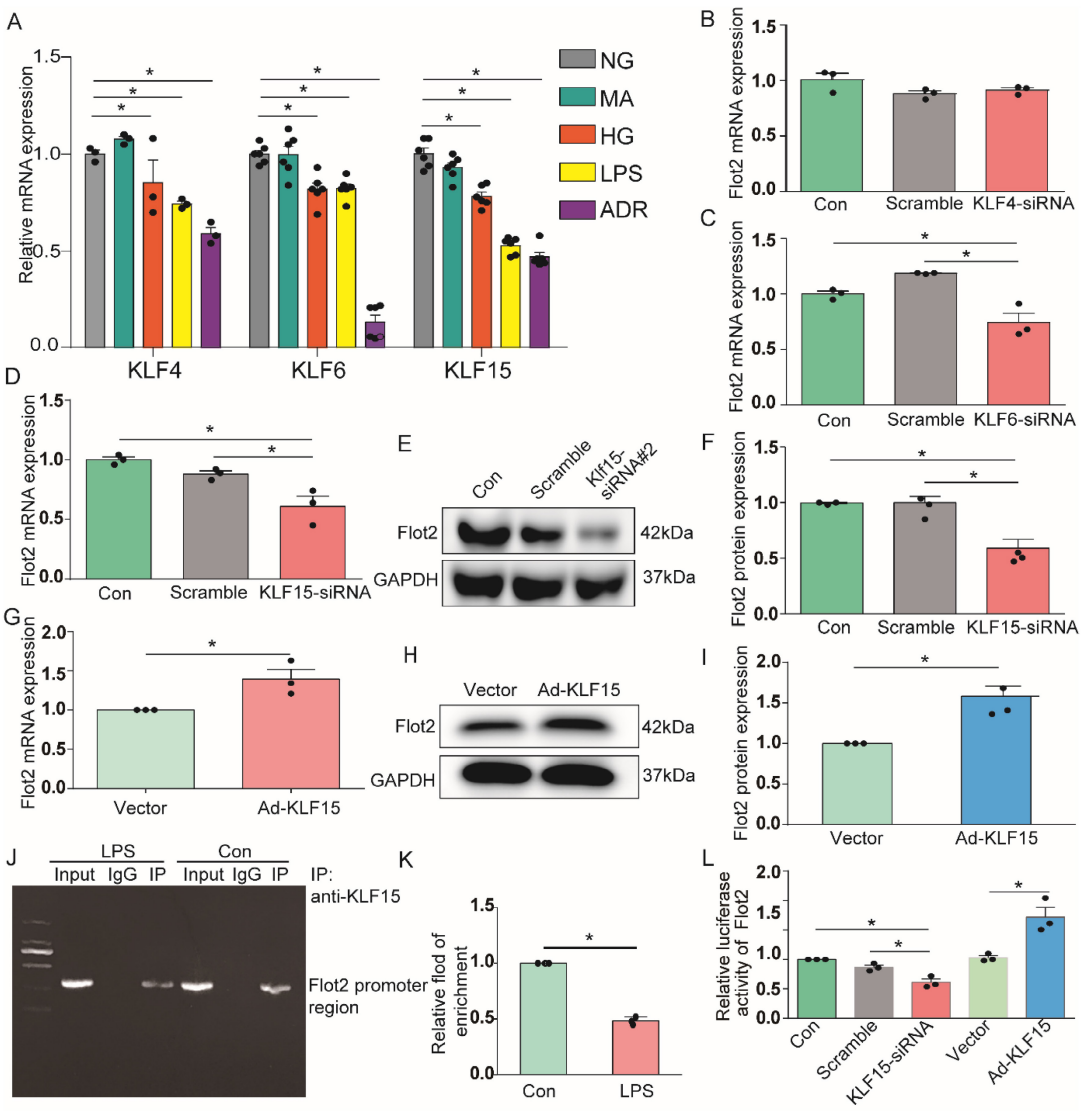
** KLF15 directly modulated the regulation of *Flot2* gene promoter activity and expression. (A)** mRNA expression levels of the transcription factors KLF4, KLF6, and KLF15 were decreased in immortalized mouse podocytes exposed to HG, LPS,or ADR. **(B-D)** Regulatory effects of KLF4, KLF6, and KLF15 on Flot2 transcription. Flot2 mRNA expression levels were significantly decreased in podocytes after silencing of KLF6 or KLF15, but not KLF4, using specific siRNAs. **(E-F)** Flot2 protein expression was decreased in podocytes after KLF15-siRNA knockdown, as shown by western blot. Data from three independent experiments.^ *^P<0.05 versus control or scramble.** (G-I)** qPCR and western blot analysis showed successful overexpression of KLF15 in immortalized mouse podocytes.** (J-K)** ChIP assay was performed to confirm the potential KLF15 binding site in the Flot2 promoter region. IgG and input fractions were used as controls. ChIP analysis in podocytes using antibody to KLF15, followed by qPCR using a Flot2 gene promoter specific primer. The amplified promoter sequence was designed at 112-456 bp upstream of the transcription start site. DNA electrophoresis showed that KLF15 bound to the Flot2 promoter in podocytes, and that binding was reduced in LPS-treated podocytes. Similar results were obtained by ChIP-qPCR. Fold enrichment = [% (ChIP/ Input)]/[%(Negative control/Input)] (n = 3). **(L)** Effects of altered KLF15 expression on luciferase activity driven by the Flot2 promoter analyzed by dual-luciferase reporter assays. Relative Flot2 transcription was decreased or elevated following KLF15-siRNA knockdown or KLF15-overexpression, respectively, in podocytes treated with adenovirus containing GFP-KLF15. Secreted alkaline phosphatase (SEAP) luminescence was used as an internal control, and the ratio of Gaussian luciferase (Gluc) activity to SEAP was calculated for normalization. Relative transcriptional activity was converted into fold-induction above the vehicle control value (*n*-fold) (*n*=3). All assays were performed in three independent experiments. Data presented as mean ± standard error. ^*^P<0.05 versus control or scramble or vector.

Previous analysis of putative transcription factor-binding sites revealed binding sites for Krüppel-like transcription factors (KLFs) in the Flot2 promoter 30. Transcription factors KLF4, KLF6, and KLF15 were expressed in glomerular podocytes, and their expression levels were significantly decreased in injured podocytes in proteinuric glomerular diseases 31-35. In the current study, KLF4, KLF6, and KLF15 mRNA expression levels were markedly reduced in immortalized mouse podocytes exposed to HG, LPS, or ADR (Figure 9A). We further explored the potential roles of these putative KLFs by real-time RT-PCR, to analyze their regulatory effects on the transcription of Flot2.Flot2 mRNA expression was significantly decreased in podocytes after silencing of KLF6 or KLF15, but not KLF4, using specific siRNAs (Figure 9B-D). Flot2 protein expression was also markedly decreased after KLF15 knockdown, as demonstrated by immunoblotting (Figure 9E-F). We overexpressed KLF15 in cultured murine podocytes to ascertain its role in Flot2 regulation (Figure 9G-I). Flot2 mRNA and protein expression levels were significantly increased following adenovirus-mediated overexpression of KLF15 in podocytes (Figure 9G-I), suggesting that KLF15 was a key regulator of Flot2 expression.

We further explored the role of KLF15 in regulating Flot2 gene activity by chromatin immunoprecipitation-quantitative (ChIP-qPCR) assay, to identify the potential KLF15-binding site in the Flot2 promoter region. We amplified the promoter sequence from 696-834 base pairs upstream of the transcription start site. DNA electrophoresis showed that KLF15 bound to the Flot2 promoter (Figure 9J). ChIP-qPCR indicated that binding was significantly reduced in LPS-treated compared with untreated murine podocytes (Figure 9K). The relative transcriptional activity of Flot2 was also dramatically reduced in response to KLF15 knockdown (Figure 9L) and elevated in response to KLF15 overexpression according to luciferase reporter assays, indicating that KLF15 directly modulated Flot2 gene promoter activity.

## Discussion

Lipid raft microdomains are critical for normal podocyte morphology and glomerular barrier function 19,36. In this study, we identified and described the functional role of raft protein Flot2 in recruiting SD proteins podocin and nephrin into rafts and mediating podocyte injury and proteinuric glomerular disease. We showed that Flot2 recruited podocin and nephrin into lipid raft microdomains. Podocyte-specific Flot2-deletion worsened albuminuria, podocyte injury, and glomerular pathology in LPS- and ADR-induced nephropathy mouse models. Conversely, overexpression of Flot2 in podocytes *in vitro* and *in vivo* attenuated cytoskeleton disruption and counteracted the reduction of SD-associated proteins in lipid rafts. Meanwhile, podocyte injury, albuminuria, and pathologic abnormalities were prevented in podocyte-specific Flot2-overexpressing transgenic mice when challenged with LPS or ADR. Furthermore, we showed that Flot2 was a direct target of KLF15. Flot2 was thus required to recruit SD proteins into rafts, and mediated podocyte injury and proteinuric glomerular disease.

Lipid rafts function as physical platforms for molecules involved in a variety of biological processes. Flot2, also known as reggie-1, was first identified in a screen for proteins involved in neuronal regeneration in goldfish 20. Flot2 belongs to the SPFH protein superfamily, members of which share an N-terminal SPFH domain, with a similar sequence but unknown function 37. In contrast to other SPFH proteins, the Flot2 C-terminus harbors a unique flotillin domain characterized by several glutamic acid and alanine repeats, which is predicted to form coiled coil structures 38,39. Flot2, as a key component of lipid rafts, belongs to the flotillin family of proteins which is highly conserved in evolution 40. Flot2 protein is ubiquitously expressed and serve important functions in a number of biological processes, including cell adhesion and migration, apoptosis, actin reorganization, and signaling transduction 41,42. The current results showed that Flot2 expression was significantly decreased in response to podocyte injury induced by LPS, ADR, and HG. Flot2 was also downregulated in renal biopsies from patients with proteinuric glomerular diseases, including MCD, FSGS, IgAN, MN, and DN. Furthermore, Glomerular Flot2 protein expression was positively correlated with eGFR and negatively correlated with proteinuria in proteinuric glomerular diseases, suggesting that Flot2 downregulation may be a common feature of podocytopathies, and that Flot2 may be a potential therapeutic target in these diseases.

We further assessed the potential roles of Flot2 in proteinuria and kidney injury by determining if reduced Flot2 expression itself could cause proteinuria, using mice with podocyte-specific targeted deletion of Flot2. Reduced expression of Flot2 caused minimal phenotypic changes at baseline, but aggravated LPS- and ADR-induced podocyte dysfunction and mesangial hyperplasia and proteinuria. Transgenic restoration of Flot2 expression in injured podocytes in the LPS- and ADR-induced proteinuric models attenuated foot process effacement, mesangial hyperplasia, and proteinuria, suggesting that Flot2 overexpression protected against podocyte injury. The murine models of podocyte injury used in our study revealed that Flot2 deficiency increased the susceptibility to podocyte injury.

Concerning the mechanism by which Flot2 regulates the podocyte phenotype, we focused on its effects on SD proteins in lipid rafts, because these proteins regulate podocyte cytoskeletal dynamics, cell survival, and glomerular filtration function 6,7. Lipid raft microdomains in podocytes form a specialized plasma membrane with a unique lipid composition. They are critical for the dynamic functional organization of the SD, and have been suggested to be important for the function of the glomerular filter 9,10,14,17,43-45. The structure of lipid rafts is dynamic, with ever-changing contents of lipids and proteins. By compartmentalizing cell membranes, the rafts recruit and cluster membrane proteins in a selective and dynamic fashion. Rafts have been proposed to form platforms for many important cellular processes, including exocytosis and endocytosis, cell adhesion, and signal transduction 46.

The *NPHS2* gene product, podocin, is a lipid raft-associated protein at the filtration slit 9. Podocin oligomerizes in lipid rafts and associates with the SD proteins nephrin and CD2AP 6, 9,19. Under normal physiological conditions, podocin and nephrin form a complex in the lipid raft. Mutations in the* NPHS2* gene disrupt nephrin targeting to lipid raft microdomains, leading to proteinuria and rapid progression to end-stage renal disease 19. These findings highlight the critical role of podocin-mediated nephrin targeting into rafts in the regulation of glomerular permselectivity. However, the exact mechanism of podocin localization in lipid rafts in podocytes, and its association with specialized lipid raft proteins, is unclear. In the current study, reducing Flot2 expression in podocytes *in vitro* and* in vivo* using siRNA and podocyte-specific Flot2-knockout mice resulted in cytoskeleton disruption and failure in recruiting the SD-associated proteins podocin and nephrin into rafts. Conversely, overexpression of Flot2 in podocytes attenuated cytoskeleton disruption and counteracted the reduction of SD proteins in lipid rafts, suggesting a role for Flot2 in recruiting these SD proteins into lipid rafts and mediating podocyte injury. A recent study also found that Flot2-mediated turnover of nephrin within the slit diaphragm is needed to maintain filter permeability in Drosophila nephrocytes 47. This will further support the role of Flot2 in recruiting these SD proteins into lipid rafts and maintainning filtration barrier permeability.

Podocin is a stomatin protein family member with a predicted hairpin-like structure localized to the insertion site of the SD in podocytes. Podocin is perhaps the most specialized member of the SPFH family, being expressed exclusively in the SD in podocytes in the kidney 9, 19, 48. Like flotillins and stomatin, podocin associates with lipid rafts. Podocin-nephrin complexes are linked to the cytoskeleton via the adaptor protein CD2AP, which binds directly to actin 9. Mutations of the podocin, nephrin, or CD2AP genes all cause severe proteinuria, emphasizing the physiological importance of podocin-based microdomains 49-51. Based on the roles of Flot2 as a key component of lipid rafts and in recruiting SD proteins to these rafts, we explored the association between Flot2 and podocin in podocytes *in vitro* and *in vivo.* Flot2 and podocin combined in podocytes *in vitro*, as shown by endogenous reciprocal co-immunoprecipitation, and similar results were obtained by reciprocal co-immunoprecipitation experiments on renal cortex lysates from *C57BL* mice. Furthermore, His- and GST-pull-down assays confirmed this direct interaction. We further investigated the protein domain responsible for this interaction by exogenous co-immunoprecipitation and GST pulldown assay, and showed that aas 30-184 of Flot2 formed a complex with aas 128-284 of podocin in podocytes. Overall, these results indicate that Flot2 interacts directly with podocin in podocytes.

Flot2 protein expression was markedly reduced in response to podocyte injury *in vitro* and *in vivo*, but the mechanism responsible for this reduction remains unclear. Flot2 mRNA expression in podocytes was decreased by different injuries, suggesting possible inhibition of its transcription. Previous analysis of putative transcription factor-binding sites revealed binding sites for KLFs in the Flot2 promoter region 30. KLFs comprise a group of 18 zinc-finger DNA-binding transcription factors involved in regulating many physiological processes, including cell proliferation, differentiation, growth, migration, apoptosis, and death 52. Regarding the kidney, recent studies showed that KLF2, KLF4, KLF5, KLF6, and KLF15 were distributed in glomeruli and renal tubules and played important roles in the occurrence and development of proteinuric glomerular diseases and acute kidney injury 28-32,53,54. KLF4, KLF6, and KLF15 were also shown to be expressed in glomerular podocytes, and their protein expression levels were significantly reduced in injured podocytes in proteinuria glomerular diseases 31-35. Flot2 promoter analysis revealed binding sites for the transcription factors KLF4, KLF6, and KLF15 in the promoter upstream 2000 bp region of the Flot2 gene. Further experiments found that Flot2 mRNA expression was significantly decreased by silencing KLF15 or KLF6 expression in podocytes *in vitro,* especially KLF15. These results were confirmed by western blotting after silencing KLF15.

To clarify the role of KLF15 in Flot2 regulation, we overexpressed KLF15 in cultured murine podocytes and revealed that Flot2 protein expression was significantly increased following adenovirus-mediated overexpression of KLF15, suggesting that KLF15 acted as a key regulator of Flot2 expression. We also explored the direct role of KLF15 in regulating* Flot2* gene promoter activity by ChIP-qPCR assay, which confirmed the potential KLF15-binding site 112-456 bp upstream of the transcription start site in the Flot2 promoter. Binding was significantly reduced in LPS-treated compared with untreated murine podocytes, while luciferase reporter assays confirmed that Flot2 transcriptional activity was dramatically reduced in response to KLF15 knockdown (Figure 9L) and elevated by KLF15 overexpression, indicating that KLF15 directly modulated Flot2 gene promoter activity.

KLF15 is a kidney-enriched nuclear transcription factor previously shown to mediate renal physiological processes and pathologic progression of CKD, involving podocyte differentiation, tubular physiology, mesangial pathology, and renal fibrosis 34,35,54. Recent evidence implicated KLF15 in mediating the salutary effects of glucocorticoids in podocytes 35,54. In addition, loss of KLF15 expression in podocytes was strongly correlated with a lack of responsiveness to glucocorticoids in human MCD and primary FSGS 38. The current study showed that KLF15 mediated the regulation of the lipid-raft protein Flot2, which was required to recruit SD proteins into the rafts, and which mediated podocyte injury and proteinuric glomerular disease. The results of this study thus not only clarify the role of KLF15 in podocyte injury in CKD, but also provide a novel rationale for the use of glucocorticoids in proteinuric glomerular disease.

To the best of our knowledge, this study provides the first evidence for the effects of the lipid-raft protein Flot2 on podocyte phenotype in the kidney, and for the role of the podocin-nephrin complex localized in lipid rafts. We found that Flot2 was widely downregulated in glomerular podocytes from patients with different glomerular diseases, and also in cultured podocytes in response to injury stimulation. We also demonstrated that Flot2 protein expression was positively correlated with eGFR and negatively correlated with proteinuria in proteinuric glomerular diseases. Reducing Flot2 expression *in vitro* and *in vivo* caused cytoskeleton disruption and failure in recruiting the SD proteins podocin and nephrin into rafts. Podocyte-specific Flot2 deletion exacerbated albuminuria, podocyte injury, and glomerular pathology in LPS- and ADR-induced nephropathy mouse models. Conversely, overexpression of Flot2 in podocytes *in vitro* attenuated cytoskeleton disruption and counteracted the reduction of SD-associated proteins in lipid rafts. Meanwhile, podocyte injury, albuminuria, and pathologic abnormalities were prevented in podocyte-specific *Flot2-*overexpressing transgenic mice challenged with LPS or ADR. Furthermore, Flot2 and podocin interacted directly with each other via their SPFH domain. We also showed that Flot2 was a direct target of the transcription factor KLF15.

**Figure 10:**
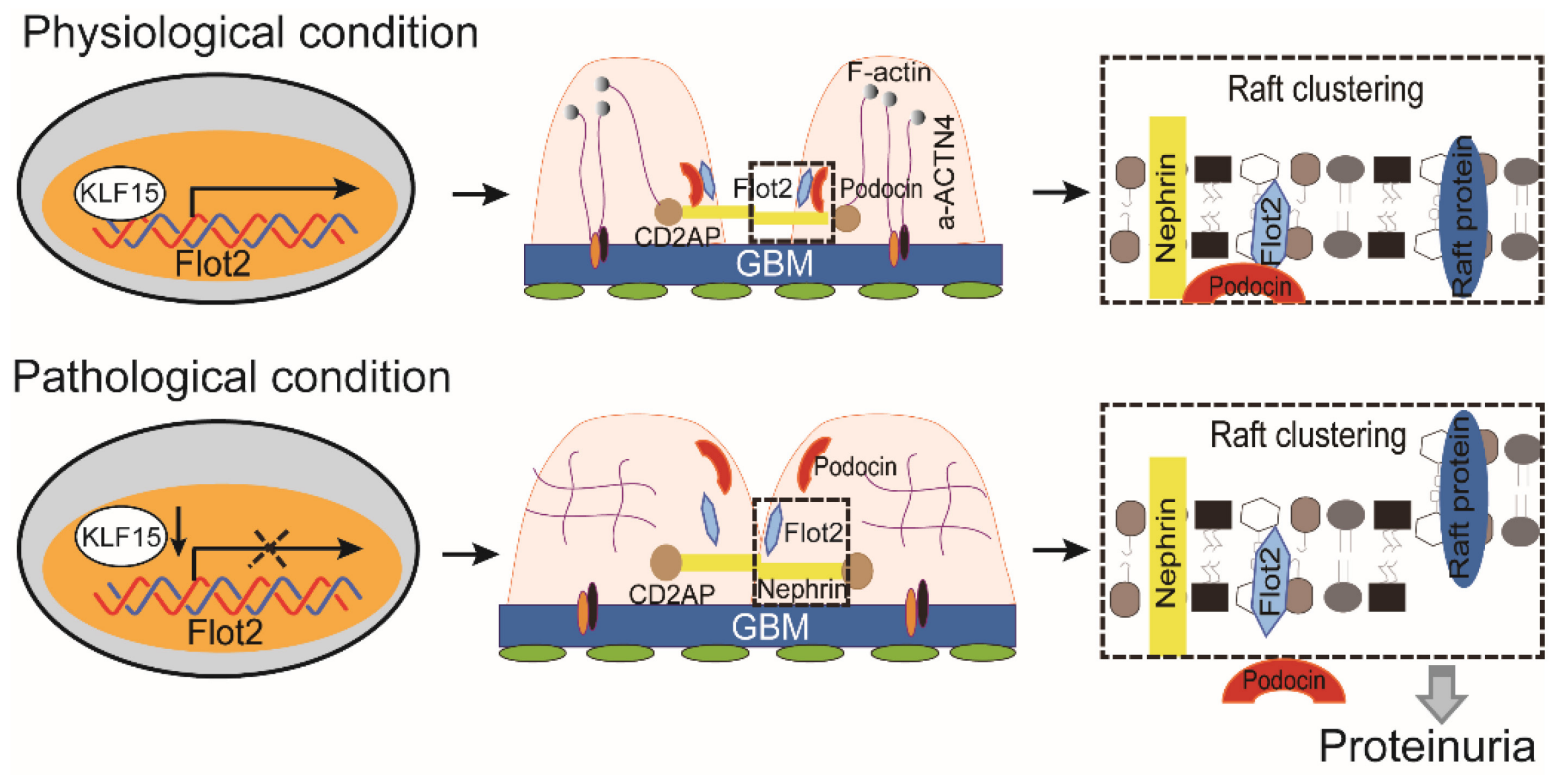
The proposed mechanism for Flot2 recruits slit diaphragm protein into rafts and mediates podocyte injury and proteinuric glomerular disease.

Collectively, these data demonstrated that Flot-2 is vital to recruit podocin-nephrin complex into rafts and attenuates podocyte injury and proteinuric glomerular disease (Figure 10). These findings highlight the importance of developing a greater understanding of interactions of lipid raft proteins in the glomerular slit diaphragm as it relates to nephrotic syndromes and shed light on what we believe are novel therapeutic strategies, maintaining necessary Flot-2 would be one potent way to prevent proteinuria kidney diseases.

## Supplementary Material

Supplementary figures and tables.
