# Supplementary material for: Flot2 acts as a novel mediator of podocyte injury in proteinuric kidney disease
Source: Int J Biol Sci. 2023 Jan 1;19(2):502–20. doi: 10.7150/ijbs.78945 (PMC9830511; doi:10.7150/ijbs.78945)
Supplement: Supplementary file 1 — Supplementary figures and tables. [file ijbsv19p0502s1.pdf]

Supplementary Detailed Methods

Table S1 The siRNA sequences that target Flot2, KLF4,KLF6 and KLF15

Table S2 Details of primers used for Real-time RT-PCR

Table S3 Forward and reverse primer sequences for the mice flot2 promoter region

Table S4 The clinical data for patients with chronic kidney disease and subjects with renal carcinoma

Figure S1 KLF4, KLF6 and KLF15 was respectively knocked down using specific interfering RNAs

Figure S2 Renal cortical expression of Flot2 in proteinuric patients. **(A)** Representative photomicrographs of Flot2 staining in adjacent normal renal cortical tissues from subjects with renal carcinoma (n=3) and from patients with MCD (n=7), FSGS (n=8), MN (n=8), IgAN (n=5), and DN (n=6). **(B)** Relative Flot2 protein expression levels in renal biopsies from different patients. **(C)** Negative correlation between Flot2 protein levels and proteinuria in all studied subjects with glomerular disease. **(D)** Positive correlation between Flot2 levels and eGFR in all studied subjects with glomerular disease. $^{*}P<0.05$ versus normal subjects.

Figure S3 Coimmunoprecipitation of endogenously expressed proteins in renal cortex lysates from C57BL mice. **(A)** Coimmunoprecipitation experiments were carried out on renal cortex lysates from C57BL mice with an antibody against Flot2. Detection of podocin in a precipitate prepared using an antibody against Flot2 but not in one prepared with an IgG. **(B)** Coimmunoprecipitation experiments were carried out on renal cortex lysates from C57BL mice with an antibody against podocin. Detection of

Flot2 in a precipitate prepared using an antibody against podocin but not in one prepared with an IgG.

Supplement Table S1 The siRNA sequences that target Flot2, KLF4, KLF6 and KLF15.

Symbol	siRNA sequence
si-m-Flot2_001	GGATGAAGCTTAAGGCTGA
si-m-Flot2_002	CAAGGATGTTTATGACAAA
si-m-Flot2_003	GGACTCTGACTGTAGAACA
si-m-Klf4_001	GCAGCTTGCAGCAGTAACA
si-m-Klf4_002	CGGAGTTGGACCCAGTATA
si-m-Klf4_003	CTATGCAGGCTGTGGCAAA
si-m-Klf6_001	CCCACTGTGACAGGTGTTT
si-m-Klf6_002	GGTGAAGTCTTAGTCAATT
si-m-Klf6_003	CCGTCCCTGGAGGAATATT
si-m-Klf15_001	CCTCAAAGTTTGTGCGAAT
si-m-Klf15_002	GCAGCAAGATGTACACCAA
si-m-Klf15_003	CCGTGTGCGAGAAGAAATT

Supplement Table S2 Details of primers used for Real-time RT-PCR

Gene	Primers
Flot2	F:5’- CGCTGTGAGGACGTAGAGAC-3’
	R: 5’- GCAGCACGACGTTCTTAATGT-3’
KLF4	F:5’- GTGCCCCGACTAACCGTTG-3’
	R:5’- GTCGTTGAACTCCTCGGTCT-3’
KLF6	F:5’- GTTTCTGCTCGGACTCCTGAT-3’
	R: 5’-TTCCTGGAAGATGCTACACATTG-3’
KLF15	F: 5’- GAGACCTTCTCGTCACCGAAA-3’
	R: 5’- GCTGGAGACATCGCTGTCAT-3’
GAPDH	F 5’- AGGTCGGTGTGAACGGATTTG-3’
	R 5’- TGTAGACCATGTAGTTGAGGTCA-3’

Note. F, forward primer; R, reverse primer.

Supplement Table S3 Forward and reverse primer sequences for the mice flot2 promoter region.

Flot2 Promoter region	Sequences
Flot2	F:TTGGCAGGGCTTGCACCTGG<br>R: CAGAAACTGTGGTTTTCTCA

Note. F, forward primer; R, reverse primer.

Supplement Table S4 The clinical data for patients with chronic kidney disease and subjects with renal carcinoma

Renal biopsy (No.)	Gender (1-male, 2-female)	Age (Years)	Diagnosis	Flot2/ Glumerulor area	24-hour urine protein	Serum creatinine (μmol/l)	eGFR(ml/min 1.73^2,CKD-EPI)
KB1923644	1	16	MCD	0.019722	9626.57	80.15	124.89
KB1925443	1	17	MCD	0.013995	12837.60	103.00	91.57
KB1922803	1	19	MCD	0.007760	9922.64	150.00	57.32
KB1919827	1	17	MCD	0.011652	9403.25	53.64	147.14
KB1922801	1	41	MCD	0.010949	7835.65	63.70	115.83
KB1922802	1	18	MCD	0.022810	6264.79	68.88	131.84
KB1918851	1	16	MCD	0.020148	10136.87	43.59	161.36
KB1926756	1	19	FSGS	0.009931	9758.78	98.00	95.90
KB1923945	1	24	FSGS	0.026889	1003.97	83.00	113.18
KB1925449	1	70	FSGS	0.018947	1647.11	114.43	55.57
KB1923125	1	38	FSGS	0.009633	5021.70	117.27	67.54
KB1920365	2	66	FSGS	0.001832	9293.45	67.41	81.67
KB1920367	2	65	FSGS	0.001148	22745.10	263.69	15.81
KB1919824	2	49	FSGS	0.006001	2205.19	55.20	105.97
KB1924493	2	61	FSGS	0.011343	7494.90	75.62	73.62
KB1926757	1	72	MN	0.006355	5597.66	99.15	65.16
KB1923940	2	72	MN	0.002803	5332.89	50.94	92.58
KB1926372	1	50	MN	0.002470	10936.90	78.77	99.65
KB1924888	1	65	MN	0.015485	989.10	62.89	98.38
KB1925444	1	35	MN	0.001215	5498.85	63.77	120.76
KB1924490	1	56	MN	0.006925	14003.45	69.61	100.51
KB1924489	1	40	MN	0.002587	4017.50	74.20	109.56
KB1927712	1	51	MN	0.005519	11444.14	176.93	37.50
KB1923933	2	37	IgA N	0.008104	6270.89	146.26	39.25

KB1926366	1	51	IgA N	0.015407	1315.73	87.60	87.72
KB1925467	2	27	IgA N	0.013634	238.04	51.51	126.53
KB1923643	1	52	IgA N	0.013220	1234.89	102.69	71.88
KB1926368	2	35	IgA N	0.024364	609.32	56.58	115.98
KB1923647	2	58	DN	0.006960	1935.54	102.69	51.94
KB1921452	1	44	DN	0.012223	4483.59	177.97	39.11
KB1917878	2	42	DN	0.014441	440.02	276.08	17.58
KB1914113	2	45	DN	0.001178	3665.78	130.90	42.43
KB1923938	1	67	DN	0.008389	10004.45	109.10	60.12
KB1914118	1	48	DN	0.008036	3208.07	190.30	35.07
ANRT	1	63	RCC	0.096561	PRO(-)	76.40	92.10
ANRT	2	57	RCC	0.114769	PRO(-)	38.80	112.50
ANRT	1	64	RCC	0.107232	PRO(-)	78.10	90.63

Note. MCD, Minimal change disease; FSGS, Focal segmental glomerulosclerosis; MN, Membranous nephropathy; IgAN, IgA nephropathy; DN, Diabetic nephropathy; ANRT, Adjacent normal renal tissue; RCC, Renal cell carcinoma

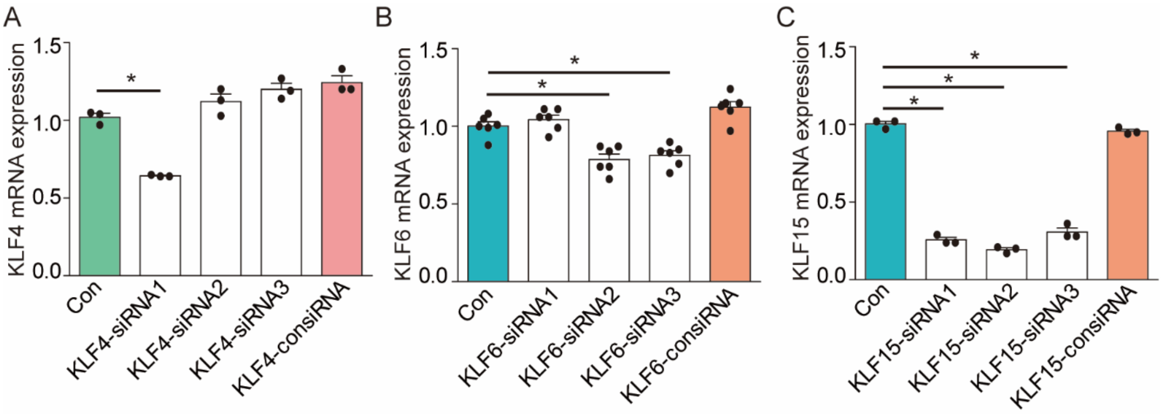

Figure S1 KLF4、KLF6 and KLF15 was respectively knocked down using specific interfering RNAs

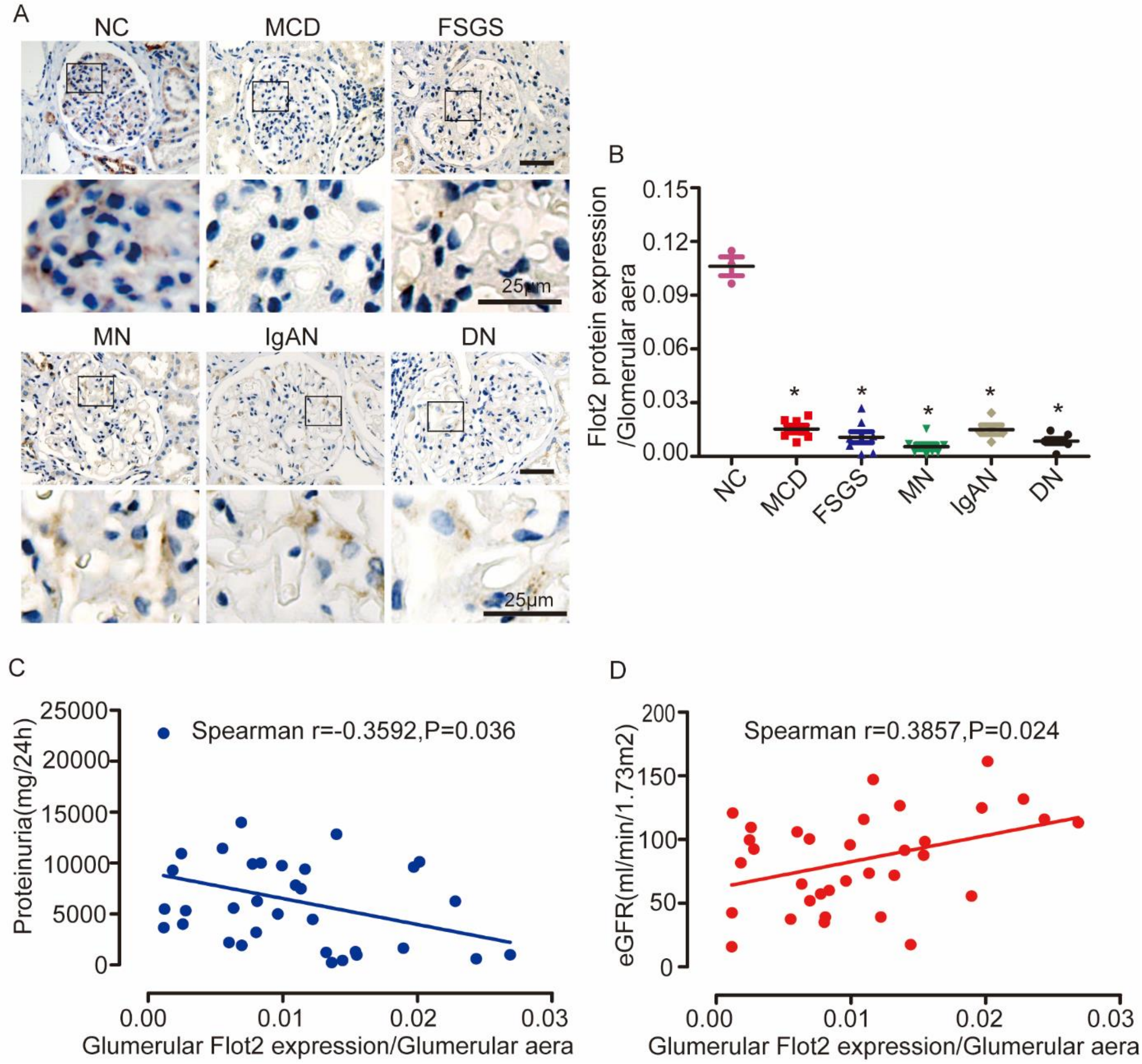

Figure S2 Renal cortical expression of Flot2 in proteinuric patients. **(A)** Representative photomicrographs of Flot2 staining in adjacent normal renal cortical tissues from subjects with renal carcinoma (n=3) and from patients with MCD (n=7), FSGS (n=8), MN (n=8), IgAN (n=5), and DN (n=6). **(B)** Relative Flot2 protein expression levels in renal biopsies from different patients. **(C)** Negative correlation between Flot2 protein levels and proteinuria in all studied subjects with glomerular disease. **(D)** Positive correlation between Flot2 levels and eGFR in all studied subjects with glomerular disease. $^{*}P<0.05$ versus normal subjects.

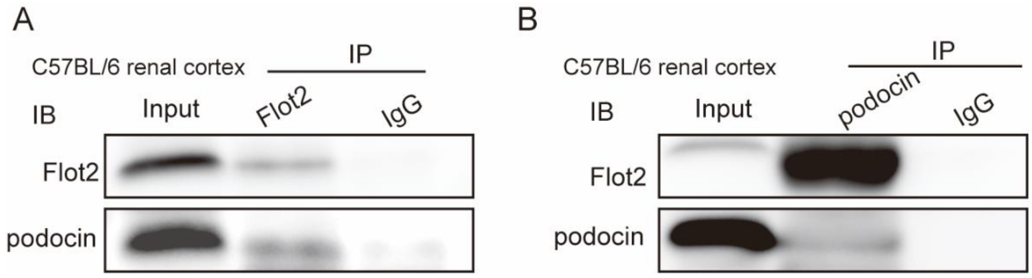

Figure S3 Coimmunoprecipitation of endogenously expressed proteins in renal cortex lysates from C57BL mice. **(A)** Coimmunoprecipitation experiments were carried out on renal cortex lysates from C57BL mice with an antibody against Flot2. Detection of podocin in a precipitate prepared using an antibody against Flot2 but not in one prepared with an IgG. **(B)** Coimmunoprecipitation experiments were carried out on renal cortex lysates from C57BL mice with an antibody against podocin. Detection of Flot2 in a precipitate prepared using an antibody against podocin but not in one prepared with an IgG.
